# Neoepitope targets of tumour-infiltrating lymphocytes from patients with pancreatic cancer

**DOI:** 10.1038/s41416-018-0262-z

**Published:** 2018-10-31

**Authors:** Qingda Meng, Davide Valentini, Martin Rao, Carlos Fernández Moro, Georgia Paraschoudi, Elke Jäger, Ernest Dodoo, Elena Rangelova, Marco del Chiaro, Markus Maeurer

**Affiliations:** 1 Department of Laboratory Medicine (LABMED), Division of Therapeutic Immunology (TIM), Karolinska Institutet, Stockholm, Sweden; 2 Centre for Allogeneic Stem Cell Transplantation (CAST), Karolinska University Hospital Huddinge, Stockholm, Sweden; 3 Department of Laboratory Medicine (LABMED), Division of Pathology, Karolinska Institutet, Stockholm, Sweden; 4 Krankenhaus Nordwest, Division of Oncology and Haematology, Frankfurt, Germany; 5 Department of Clinical Science, Pancreatic Surgery Unit, Division of Surgery, Intervention and Technology (CLINTEC), Karolinska Institutet, Stockholm, Sweden; 6 Present Address: Department of Oncology/Haematology, KHNW, Frankfurt, Germany & ImmunoSurgery Unit, Champalimaud Foundation, Lisbon, Portugal

## Abstract

**Background:**

Pancreatic cancer exhibits a poor prognosis and often presents with metastasis at diagnosis. Immunotherapeutic approaches targeting private cancer mutations (neoantigens) are a clinically viable option to improve clinical outcomes.

**Methods:**

3/40 TIL lines (PanTT26, PanTT39, PanTT77) were more closely examined for neoantigen recognition. Whole-exome sequencing was performed to identify non-synonymous somatic mutations. Mutant peptides were synthesised and assessed for antigen-specific IFN-γ production and specific tumour killing in a standard Cr51 assay. TIL phenotype was tested by flow cytometry. Lymphocytes and HLA molecules in tumour tissue were visualised by immunohistochemistry.

**Results:**

PanTT26 and PanTT39 TILs recognised and killed the autologous tumour cells. PanTT26 TIL recognised the KRAS_G12v_ mutation, while a PanTT39 CD4^+^ TIL clone recognised the neoepitope (GLLR**Y**WRTERLF) from an aquaporin 1-like protein (gene: *K7N7A8*). Repeated stimulation of TILs with the autologous tumour cells line lead to focused recognition of several mutated targets, based on IFN-γ production. TILs and corresponding PBMCs from PanTT77 showed shared as well as mutually exclusively tumour epitope recognition (TIL-responsive or PBMC-responsive).

**Conclusion:**

This study provides methods to robustly screen T-cell targets for pancreatic cancer. Pancreatic cancer is immunogenic and immunotherapeutic approaches can be used to develop improved, targeted therapies.

## Background

The World Health Organization (WHO) reported that pancreatic cancer claimed >330,000 lives in 2012, with 68% of deaths occurring in countries with a high to very high human development index (HDI).^1^ Since most patients present with metastatic disease at diagnosis, the 5-year survival rate is a meagre 5%. These statistics commensurate with limited treatment options for patients with pancreatic cancer, which include surgery (only 10–20% of patients qualify for this option) or chemotherapy with gemcitabine, sometimes in combination with the tyrosine kinase inhibitor erlotinib.^2^ A regimen of gemcitabine plus nab-paclitaxel (Abraxane^®^) improves overall and progression-free survival in patients with locally advanced or metastatic pancreatic cancer compared to gemcitabine alone, albeit with treatment-related toxicities.^3,4^ The combination regimen of oxaliplatin, irinotecan, fluorouracil and leucovorin (FOLFIRINOX) lead to improved progression-free survival of patients compared to gemcitabine alone (median of 6.4 months versus 3.3 months).^5,6^ Due to toxicity (grades 3–4) related to the use of FOLFIRINOX, this regimen is recommended for younger patients.^5,7^

Cancer immunotherapy is a progressively expanding discipline within modern oncology. Immune-based treatment of metastatic melanoma using interleukin (IL) 2-conditioned autologous lymphocytic cells from patients’ blood has existed since the 1980s.^8–11^ Monoclonal antibody-based blockade of the immune checkpoint inhibitors programmed cell death protein 1 (PD-1) and cytotoxic T lymphocyte-associated antigen 4 (CTLA-4) has further revolutionised the field. The clinical efficacy of PD-1 blockade in patients with metastatic cancer appears to depend on activation of T-cell populations with T-cell receptors (TCRs), which specifically recognise mutated host molecules, ie, neoepitopes.^12^ These mutant proteins harbouring point mutations, termed neoepitopes, represent a clinically significant step towards refining T-cell-based immunotherapies and cancer vaccines. Neoepitopes differ from one patient to another (individual ‘mutanome’), giving rise to a highly personalised antigen signature. In line with this, specialised T cell-based therapies targeting private mutations in patients with metastatic cancers have resulted in remarkable clinical responses.^13–15^

We have previously reported that tumour-infiltrating lymphocytes (TILs) can be reliably and successfully isolated from pancreatic cancer lesions and expanded in vitro using a cocktail of IL-2, IL-15 and IL-21.^16^ In the present study, we characterised the neoepitope recognition profile of TILs as well as peripheral blood T cells from patients with pancreatic cancer.

## Materials and methods

### Patient characteristics

Pancreatic tumour samples from three patients with pancreatic cancer were obtained as part of a clinical research project approved by the Regional Ethical Review Board (Regionala etikprövningsnämnden) at Karolinska Institutet, Sweden (EPN: 2013/1332-31/3 and 2013/977-31/1). The clinical characteristics of the patients are presented in Table 1.

**Table 1:** Clinical characteristics and HLA profile of patients with pancreatic cancer

*Clinical characteristics*												
Patient ID	Age	Gender	Tumour tissue source				Histology			Survival after tumour sampling (days)		
PanTT26	61	Male	Biopsy				Pancreatic ductal adenocarcinoma			547		
PanTT39	62	Female	Surgery				Adenocarcinoma of pancreatobiliary type			411		
PanTT77	68	Male	Biopsy				Pancreatic ductal adenocarcinoma			355		

*HLA type of the patients*												
Patient ID	HLA-A	HLA-A	HLA-B	HLA-B	HLA-C	HLA-C	HLA-DRB1	HLA-DRB1	HLA-DQA1	HLA-DQA1	HLA-DQB1	HLA-DQB1
PanTT26	30:02	68:01	27:05	39:01	05:01	12:03	03:01	13:01	01:03	05:01	02:01	06:03
PanTT39	01:01	02:01	08:01	51:01	01:02	07:01	01:01	03:01	01:01	05:01	02:01	05:01
PanTT77	24:02	26:01	07:02	44:02	05:01	07:02	15:01	15:01	01:02	01:02	06:02	06:02

### Generation of TILs and autologous tumour cell lines

Pancreatic cancer TILs and autologous tumour cell lines were generated as previously described.^16,17^ Tumour cells required for the cytotoxicity experiments were obtained during passage 15–20.

### PBMC isolation

Peripheral blood mononuclear cells (PBMCs) were isolated from whole blood (PanTT77) over a Ficoll-Hypaque gradient (GE Healthcare, Uppsala, Sweden) and washed twice in sterile PBS prior to use in experiments.

### DNA isolation, whole-genome sequencing, mutanome analysis and neoepitope synthesis

Isolation and purification of genomic DNA, library construction, exome capture of all coding genes as well as next-generation sequencing of tumour tissue and control patient samples (TILs) were performed as previously described by Jones et al.^18^. Briefly, genomic DNA from patient samples (tumour tissue and TILs) was fragmented for constructing an Illumina DNA library (Illumina, San Diego, CA). Regions of DNA corresponding to exons were captured in solution using the Agilent SureSelect 50 Mb kit Version 3 as per manufacturer’s instructions (Agilent, Santa Clara, CA). Paired-end sequencing resulting in 100 bases from each end of every fragment was performed using a HiSeq 2000 Genome Analyser (Illumina). Results of the sequencing data were mapped to the reference human genome sequence. Alterations within the sequencing data were determined by comparing over 50 million bases of tumour DNA from non-malignant lesions. A high fraction of the sequences obtained for each sample was found to occur within the captured coding regions. More than 43 million bases of target DNA were analysed in the tumour and normal samples; an average of 42–51 reads per base was obtained for both sample types. The tags were aligned to the human genome reference sequence (hg18) using the Eland algorithm of CASAVA 1.6 software (Illumina). The chastity filter of the BaseCall software of Illumina was used to select sequence reads for subsequent analysis. The ELANDv2 algorithm of CASAVA 1.6 software (Illumina) was applied for identifying point mutations, small insertions, deletions or stop codons in the sequences obtained. Mutation polymorphisms recorded in the Single Nucleotide Polymorphism Database (dbSNP) were excluded from analysis. Potential somatic mutations were filtered out as previously described,^18^ while only non-synonymous single and dinucleotide substitutions, respectively, were listed in an Excel spreadsheet for downstream work. The filter criterion for selecting candidate peptides is that the expression level of mutated genes in tumour tissue surpasses 5%. Alternatively spliced products or mutated sequences with stop codons may result in epitopes that are shorter than the standard 15-mer peptides that are used for screening immunogenicity. The length of the resulting peptide sequences was set at 15-mer to include all possible epitopes presented by HLA class I (8–10 amino acids) as well as HLA class II (11–20 amino acids) molecules.

After identification of mutations through whole-exome sequencing followed by in silico analysis, the 15-mer peptides were constructed by placing the mutation at the centre position of the 15-amino acid sequence (Peptide & Elephants, Berlin, Germany). The corresponding wild-type epitopes were also synthesised to compare the matched mutant and wild-type sequences (peptide pairs) in immunological assays.

### Evaluation of the immunoreactivity of TILs to neoepitopes

TILs or PBMCs (1.0 × 10^5^ cells) were cultured in 200 μl of T-cell medium with 1 μg of the individual wild-type or mutated peptide in round-bottom 96-well microtiter plates. Negative controls contained assay medium alone while the positive control contained 30 ng/mL of the anti-human CD3 antibody clone OKT3 (Biolegend, San Diego, CA) for maximal TCR stimulation. Cells were incubated for 3 days at 37 °C with 5% CO_2_, after which supernatants were harvested for interferon (IFN)-γ production using a standard sandwich enzyme-linked immunosorbent assay (ELISA) kit (Mabtech, Stockholm, Sweden). Values from the negative control (medium) were subtracted from epitope (peptide)-specific responses and the data reported to reflect the IFN-γ production (in pg/3 days/1.0 × 10^5^ TILs representing the net IFN-γ production from T-cell populations). The mAbs w6/32 (anti-MHC class I, HLA-A, B and -C) and L243 (anti-HLA-DR) were used as blocking antibodies to asses MHC class I or -class I restriction.

### Repeated TIL stimulation with the autologous tumour cell line

TILs were stimulated with the autologous tumour cell line in six-well tissue culture plates (5 × 10^6^ TILs: 1 × 10^6^ tumour cells) containing T-cell medium for 7 days, after which TILs were stimulated with (autologous) tumour cells two more times.

### CD107a induction assay

The CD4^+^ TIL clone was generated by limiting dilution and confirmed using the IO Test^®^ Beta Mark TCR V beta Repertoire Kit (Beckman Coulter, Brea, CA) by flow cytometry. A total of 2 × 10^5^ T cells were co-cultured with 4 × 10^4^ autologous tumour cells for 5 h at 37 °C (and 5% CO_2_) in a 96-well tissue culture plate containing 200 μl assay medium/well (RPMI 1640 with 10% FBS and penicillin/streptomycin; both from Thermo Fisher Scientific, Waltham, MA). During the incubation period, 1.3 μg/ml of monensin (Merck KGaA, Darmstadt, Germany), and 4 μl of the anti-human CD107a-Alexa Fluor 700 antibody (Clone H4A3; BD Biosciences, Franklin Lakes, NJ) were added. PMA = phorbol 12-myristate 13-acetate (PMA) was used as the positive control and assay medium alone without tumour cells was used as negative control. After 5 h of incubation, the cells were stained with anti-human CD3-PE/Cy7 (Clone HIT3A; BioLegend, San Diego, CA), anti-human CD4-V450 (Clone RPA-T4) and anti-human CD8-APC/Cy7 (Clone SK1) (both from BD Biosciences, Franklin Lakes, NJ), and analysed by flow cytometry.

### TIL phenotype characterisation by flow cytometry

TILs were stained with anti-CD3 Brilliant violet 570, anti-CD4 Brilliant violet 510, anti-CXCR3 FITC (all from Biolegend, San Diego, CA) and anti-CD8a APC-Cy7 (BD Biosciences, Franklin Lakes, NJ). After 15 min, cells were washed in PBS-0.1% FBS, and analysed by flow cytometry. Differentiation and maturation marker analysis based on CD45RA and CCR7 expression was performed as described previously.^17^

### Flow cytometry and analysis

All flow cytometry experiments were performed on a BD FACS Aria flow cytometer, while data analysis was performed using FlowJo software version 7 (both from BD Biosciences).

### Immunohistochemistry

Resected pancreatic specimens were fixed in 4% formalin and processed for routine histopathological diagnosis. The fixed tissue blocks were then embedded in paraffin, sectioned (thickness of 4 μm) and stained with haematoxylin-eosin for light microscopy. A specialist pancreatic pathologist (Dr. C. F. Moro) chose from each specimen a tissue block in which the tumour centre, tumour border and surrounding non-tumour pancreatic tissue was represented. The selected blocks were subsequently sectioned and processed for immunohistochemistry. To preserve the relative spatial distribution of the lymphocyte populations, chromophore-conjugated antibodies were combined into the following 2-plex immunostainings: CD3-CD20 and CD4-CD8. Details of the individual antibodies are as follows: anti-CD3 (clone LN10; Leica Biosystems, Wetzlar, Germany), anti-CD20 (clone L26; Agilent Technologies, Santa Clara, CA), anti-CD4 (clone 4B12; Leica Biosystems), anti-CD8 (clone C8/144B; Agilent Technologies). Staining was performed using a Leica BOND III automated immunostainer (Leica Biosystems). The primary antibodies were detected using 3,3′-diaminobenzidine (DAB—brown) as chromogen and the secondary antibodies with alkaline phosphatase (AP—red). HLA class I and class II immunohistochemistry staining was performed separately, according to the above-mentioned protocol. Technical details of the reagents used are as follows: anti-human HLA-class I ABC antibody (clone EMR8-5) and anti-human HLA-class II DR, DP and DQ antibody (clone CR3/43) (both antibodies from Abcam, Cambridge, UK). The antibodies were detected using 3,3′-diaminobenzidine (DAB—brown).

## Results

The reliable expansion of CD4^+^ and CD8^+^ TILs from pancreatic cancer tissue using IL-2, IL-15 and IL-21, particularly within the central and effector memory compartments, is shown in Supplementary Figure 1. To better facilitate presentation of data relevant to the  current study, the results section has been organised to reflect the findings pertinent to each patient individually. The clinical characteristics of the patients are presented in Table 1.

### Patient PanTT26

TILs and the corresponding tumour cell line was established from patient PanTT26. We previously showed that CD4^+^ and double-negative (CD3^+^ ‘DN’, CD4^−^CD8^−^) T cells among TILs derived from patient PanTT26 (annotated as Panc17 in our previous publication^16^) are able to produce IL-2 and IL-17, while CD8^+^ T cells do not produce cytokines after 6 h of stimulation with autologous tumour cells.^16^ In addition, we also observed that PanTT26 TILs could kill autologous tumour cells within 4 h of culture initiation, using a standard Cr51-release assay. We, therefore, intended to further explore the anti-tumour characteristics of PanTT26 in the present study.

**Fig. 1:**
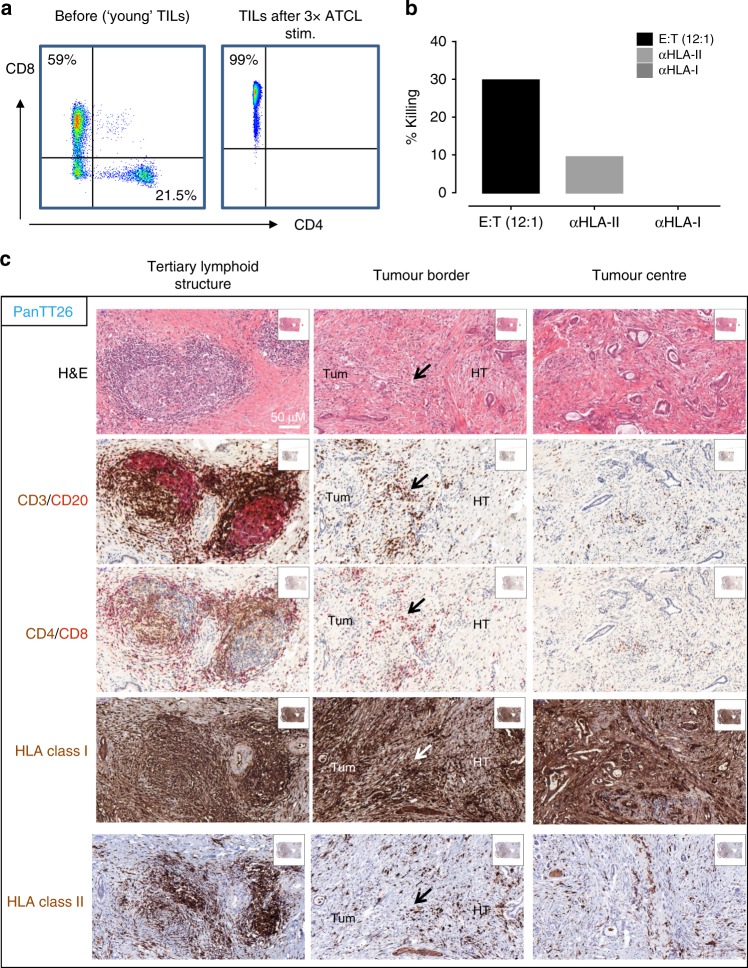
Characterisation of the TILs obtained from patient PanTT26. **a** Flow cytometric analysis revealed that TILs from patient PanTT26 comprised 60% CD8^+^ T cells. After 3× stimulation of PanTT26 TILs with the autologous tumour cell line, the CD8^+^ T-cell frequency increased to 99%. **b** TILs from patient PanTT26, prior to 3× stimulation with the autologous tumour cells, were co-incubated with the autologous tumour cell line at a ratio of 12:1 (TILs:tumour cells; represented as effector (*E*) to target (*T*) cell ratio) in a standard 4-h chromium-51 release assay. Parallel wells with the TIL:tumour cell co-culture were incubated with either anti-HLA class-I antibody (clone W6/32) or anti-HLA class-II antibody (clone L243, anti-HLA-DR) to test for decreased tumour cell killing using the blocking antibody (interfering with the MHC class I or MHC class II antigen presentation). While blockade of HLA class II antigen presentation partially reduced cytotoxicity of TILs, blockade of HLA-class I-restricted antigen presentation totally abrogated killing of the tumours by autologous TILs. This recognition experiment was performed using ‘young TIL’ representing a majority of CD8^+^ TILs still containing CD4^+^ T cells. **c** Four-micrometre sections of resected PDAC tumour tissue from patient PanTT26 were used for immunohistochemistry. Three different anatomical compartments of the diseased pancreas were assessed: the centre of the tumour mass, the tumour border as well as the tertiary lymphoid structures (TLS) adjacent to the primary tumour mass in the tissue. Haematoxylin and eosin (H&E) staining was performed as for routine analysis of the cellularity of the tumour sections; CD3 and CD20 immunostaining was performed to gain an overview of the proportions of T and B cells present in the tumour and its immediate tissue environment; immunostaining of CD4-positive and CD8-positive cells was done to visualise the major T-cell populations infiltrating the tumour mass. The TLS appears to be enriched for lymphocyte infiltration compared to the centre of the tumour mass as well as the tumour border, including a B-cell infiltrate. Expression of HLA class I and class II molecules in the tumour tissue was also performed. The PanTT26 tumour consists largely of HLA-class I-positive cancer cells and some stromal cells but none of the tumour cells stained HLA-class II positive. Importantly, there appear to be more CD8^+^ T cells than CD4^+^ T cells in the tumour border area and the centre of the tumour mass. The black arrows in the tumour border immunostaining indicate the cellular rim that separates the diseased tissue and tumour mass from healthy pancreas parenchyma. ATCL autologous tumour cell line, Tum tumour, HT healthy tissue

We observed by flow cytometry that TILs from patient PanTT26 comprised ~59% CD8^+^ T cells and 22% CD4^+^ T cells (Fig. 1a). The TILs were then stimulated with the PanTT26 tumour cell line (autologous) three times to see whether repeated exposure of the IL-2/IL-15/IL-21-conditioned TILs to the tumour would lead to enrichment of tumour epitope-reactive T cells. The resulting TILs (after 3× stimulation with autologous tumour cells) were enriched for CD8^+^ TILs (almost 100%), while CD4^+^ T cells were entirely absent (from 22% pre-stimulation to 0% post-stimulation with tumour cells) based on flow cytometric data. Using the W6/32 (anti-HLA-I) and L243 (anti-HLA-DR) antibodies in Cr51-release assays, we found that TILs, prior to 3× stimulation with the autologous tumour cell line, displayed a dampened cytotoxic effect with HLA class II inhibition, while the W6/32 (anti-MHC class I) antibody abrogated tumour recognition completely (Fig. 1b). This observation indicated that the cytotoxic effect of PanTT26 TILs was mainly restricted by HLA class I antigen presentation. Furthermore, immunohistochemistry studies revealed that the PanTT26 tumour did not express HLA-class II molecules in situ (Fig. 1c).

Whole-exome sequencing of the pancreatic tumour tissue from patient PanTT26 was performed to identify cancer-related mutations that may give rise to mutated antigens (neoantigens). Mutated peptide sequences (containing neoepitopes) were predicted by placing the mutation at the centre of the sequence and flanking it with seven amino acids on either side. The resulting peptide sequences were synthesised along with the corresponding wild-type sequences and tested for T-cell reactivity with TILs by measuring antigen-specific IFN-γ production in the culture supernatants by sandwich ELISA. In total, 298 peptides (149 wild type and mutated, respectively) were tested for both ‘young’ TILs and TILs that were stimulated three times with the autologous tumour cells. We found that >150 pg IFN-γ (per 10*e*5 T cells/1 μg peptide) was produced by young TIL or tumour cell-stimulated TIL in response to subsequent exposure to wild-type or mutated peptides (Table 2A). A more extensive list of these results is shown in the Supplementary Table 1.

**Table 2A:** Peptide-specific IFN-γ production by PanTT26 and PanTT39

				IFN-γ (pg/10*e*5 TIL/1 μg peptide			
				Young TIL		Stimulated three times with tumour	
Peptide ID	Wild-type sequence	Mutated sequence	Gene name	Wild type	Mutated	Wild type	Mutated
PanTT26-P1	FEGTEMWNPNRELSE	FEGTEMWYPNRELSE	*ACHE*	104	14	228	0
PanTT26-P4	PWRKFPVYVLGQFLG	PWRKFPVHVLGQFLG	*AQP7*	152	315	0	443
PanTT26-P7	STAYPAPMRRRCCLP	STAYPAPVRRRCCLP	*ARMC7*	261	122	446	0
PanTT26-P8	VALKPQERVEKRQTP	VALKPQECVEKRQTP	*AUTS2*	94	0	167	0
PanTT26-P10	TPEPAIPPKATLWPA	TPEPAIPHKATLWPA	*C6orf132*	156	0	0	0
PanTT26-P12	THRPGGKHGRLAGGS	THRPGGKRGRLAGGS	*CCDC74B*	104	114	0	487
PanTT26-P13	VTVHPTSNSTATSQG	VTVHPTSKSTATSQG	*CD68*	41	0	0	281
PanTT26-P20	SSLPGPPGPPGPPGP	SSLPGPPGPPGPRGY	*COL18A1*	4	1	0	169
PanTT26-P23	MSYDYHQNWGRDGG	MSYDYHHNWGRDGG	*DHX36*	110	46	164	0
PanTT26-P28	LADGEGGGTDEGIYD	LADGEGGATDEGIYD	*EFS*	20	190	0	0
PanTT26-P36	KLVVVGAGGVGKSAL	KLVVVGAVGVGKSAL	*KRAS*	54	0	0	**453**
PanTT26-P37	LFGLGKDEGWGPPAR	LFGLGKDVGWGPPAR	*NT5C3B*	65	100	0	3
PanTT26-P39	MRHFCLISE	MHHFCLISE	*TMEM168*	124	0	355	0
PanTT26-P43	KPVILGVRWYVETTS	KPVILGVCWYVETTS	*KLK6*	57	16	166	0
PanTT26-P45	SSGGGSSGGGYGGGS	SSGGGSSSGGYGGGS	*KRT10*	94	19	242	0
PanTT26-P53	GRKFAAWAPPSFSQT	GRKFAAWGPPSFSQT	*PTX4*	240	94	457	0
PanTT26-P59	GYGEMGSGYREDLGA	GYGEMGSVYREDLGA	*IGFN1*	21	39	0	488
PanTT26-P60	LLDRGSFRNDGLKAS	LLDRGSFWNDGLKAS	*KALRN*	42	87	0	354
PanTT26-P61	SQLMLTRKAEAALRK	SQLMLTRKGNASCLE	*KANSL1*	196	0	74	0
PanTT26-P62	ALKIKGIHPYHSLSY	ALKIKGIRPYHSLSY	*KIAA1109*	177	139	65	0
PanTT26-P68	PRCCISSCCRPSCCV	PRCCISSFCRPSCCV	*KRTAP4-11*	56	189	0	325
PanTT26-P69	CRPQCCQSVCCQPTC	CRPQCCQTVCCQPTC	*KRTAP4-9*	101	0	279	0
PanTT26-P70	TCCRTTCYRPSCCVS	TCCRTTCFRPSCCVS	*KRTAP4-9*	217	118	67	0
PanTT26-P76	DEMDCPLSPTPPLCS	DEMDCPLRPTPPLCS	*MALRD1*	117	51	222	527
PanTT26-P81	EKKQQFRNLKEKCFL	EKKQQFRSLKEKCFL	*NBPF10*	169	212	0	164
PanTT26-P83	KLKKKQVNVFA	KLKKKQVKVFA	*NCOR1*	106	327	0	710
PanTT26-P84	GRLILWEAPPLGAGG	GRLILWEGPPLGAGG	*NEK8*	97	32	0	164
PanTT26-P93	TYSPTSPVYTPTSPK	TYSPTSPDYTPTSPK	*POLR2A*	66	0	511	0
PanTT26-P95	SKMGKWCRHCFAWCR	SKMGKWCSHCFAWCR	*POTEH*	200	176	58	149
PanTT26-P96	SKMGKWCRHCFPCCR	SKMGKWCSHCFPCCR	*POTEJ*	297	261	0	137
PanTT26-P99	NLVHGPPAPPQVGAD	NLVHGPPGPPQVGAD	*PSD*	57	97	80	328
PanTT26-P103	PSRHRYGARQPRARL	PSRHRYGTRQPRARL	*RNF126*	152	233	0	142
PanTT26-P107	AGRFGQGAHHAAGQA	AGRFGQGDHHAAGQA	*SBSN*	95	80	44	205
PanTT26-P108	QLLEGLGFTLTVVPE	QLLEGLGCTLTVVPE	*SERPINA13P*	47	2	425	0
PanTT26-P114	IHSSWDCGLFTNYSA	IHSSWDCSLFTNYSA	*TMC8*	38	61	0	408
PanTT26-P115	IMASKGMRHFCLISE	IMASKGMHHFCLISE	*TMEM168*	132	92	213	193
PanTT26-P116	LWHLQGPKDLMLKLR	LWHLQGPEDLMLKLR	*TMPRSS6*	275	12	371	0
PanTT26-P119	LGLWRGEEVTLSNPK	LGLWRGEAVTLSNPK	*TRIP12*	13	74	0	240
PanTT26-P122	RKFISLHRKALESDF	RKFISLHKKALESDF	*WDFY4*	23	249	0	562
PanTT26-P124	GCGKVFARSENLKIH	GCGKVFACSENLKIH	*ZIC1*	43	0	186	0
PanTT26-P132	SNLTKHKKIHIEKKP	SNLTKHKIIHIEKKP	*ZNF43*	369	170	703	0
PanTT26-P140	NVAKPSSGPHTLLHI	NVAKPSSCPHTLLHI	*ZNF626*	80	0	465	0
PanTT26-P146	HKRIHNGEKPYKCEE	HKRIHNGDKPYKCEE	*ZNF730*	0	137	0	298
PanTT26-P147	EKPYSCPDCSLRFAY	EKPYSCPECSLRFAY	*ZNF785*	94	15	159	0
PanTT26-P148	KCEECDTVFSRKSHH	KCEECDTDFSRKSHH	*ZNF860*	47	28	396	0

TILs before (‘young’ TILs) and after repeated stimulation with the autologous tumour cell line. Shown are T-cell responses to mutant peptide targets, defined by IFN-γ production, after 3× stimulation with the autologous tumour cell line, indicating immune epitope/TCR repertoire focusing. The full list of peptides tested for IFN-γ production is provided in Supplementary Table 1. The immune-reactivity to the mutated KRAS peptide, which was seen only after 3x stimulation with the autologous tumour cell line, is presented in bold due to the clinical significance of KRAS-directed immune reactivity in cancer and PDAC pathogenesis^19^. Furthermore, it was recently shown in the setting of metastatic colorectal cancer that T-cell responses to mutated KRAS can provide clinical benefit by inducing disease regression^15^

Increased IFN-γ production to the mutated KRAS peptide KLVVVGA**V**GVGKSAL (representing the well-described KRAS_G12V_ mutation) was observed after 3× stimulation with the autologous tumour cell-stimulated TILs compared to young TILs (Table 2A). The clinical relevance of this finding is underlined by the established knowledge that oncogenic mutant KRAS commonly plays a crucial role in PDAC pathogenesis.^19^ Garcia-Silva and Aranda have shown that thyroid hormone nuclear receptors (TRs) can repress RAS-dependent cellular transformation and tumour growth.^20^ A subsequent study from the same group showed that cellular nuclear receptor co-repressor 1 (NCOR1) levels could increase upon TR(s) expression.^21^ Intriguingly, the strongest IFN-γ response by both PanTT26 young TILs and tumour cell-stimulated TILs was observed after exposure to a mutated peptide from NCOR1 (KLKKKQV**K**VFA, mutation: N99K). Young TILs produced 327 pg IFN-γ/10*e*5 TILs in response to mutated NCOR1, while tumour cell-stimulated TILs showed 710 pg IFN-γ/10*e*5 TIL (Table 2A). We also noticed that the *NCOR1* gene product in the PanTT39 tumour harboured a mutation at position 120 (R120L).

WD repeat- and FYVE domain-containing protein 4 (WDFY4) is highly expressed in lymph nodes and the spleen; previous studies have shown that aberrations in this gene are associated with autoimmune diseases such as systemic lupus erythematosus and rheumatoid arthritis.^22,23^ However, the significance of WDFY4 in cancer is yet to be explored. PanTT26 TILs also showed strong IFN-γ responses to a mutated peptide derived from WDFY4 (RKFISLH**K**KALESDF). We noticed that 17% of mutations (25/149 mutations) in PanTT26 are associated with zinc-finger proteins (ZNF), which display diverse biological functions.^24^ The recognition of a ZNF730-derived peptide was pronounced following stimulation of PanTT26 TILs with autologous tumour cells, although four other wild-type ZNF peptides were recognised (Table 2A). It is plausible that a high number of wild-type ZNF targets were obtained due to the filter that was applied for detecting mutations in the tumour samples (minimum of 5% mutation load). Of note, ZNF3, ZNF257, ZNF479 and ZNF493, which were found to be mutated in the PanTT26 tumour, also appeared to be mutated in the PanTT39 tumour specimen. The function and immunological significance of ZNF as a target for cellular immune responses in pancreatic cancer therefore warrants further exploration.

### Patient PanTT39

TILs isolated from this patient were characterised by flow cytometry and found to contain exclusively CD4^+^ T cells (>99%) (Supplementary Figure 2). We also performed whole-exome sequencing using DNA from PanTT39 tumour tissue and generated mutated as well as the corresponding wild-type peptide sequences to gauge for T-cell reactivity. Following mutation analysis, 1447 mutations were found, as compared to 149 mutations in PanTT26 tumour, thus reflecting a 10-fold higher mutational burden in patient PanTT39. A mutation in the *BRCA1* gene product (R600L) was also identified. This is of note, since BRCA1 mutations are implicated as a key contributing factor related to the burden of somatic mutations in pancreatic cancer.^25^ We also found seven-point mutations in the HLA-A alleles, two-point mutations in the HLA-B alleles and eight-point mutations in the HLA-C alleles, which ultimately gave rise to amino acid changes in the resulting protein products associated with the HLA class I antigen processing and presentation pathway (Supplementary Table 2). Since the TIL line from PanTT39 consisted exclusively of CD4^+^ T cells and no CD8^+^ T cells, we focused on the peptides that could bind HLA class II molecules. Fourteen HLA class II-binding targets were identified using a predicted consensus rank of ≤1.0 (Supplementary Table 3). It is important to mention here that the mutational burden among HLA-DRB1 alleles in PanTT39 tumour was calculated as 8.8%. Peptides that would bind to HLA-DRB1 were nevertheless incorporated, assuming >90% chance that an adequate number of tumour cells would still be able to present antigen via HLA-DRB1. TILs from this patient were then screened for recognition of peptides in a 3-day 96-well co-culture assay, as described for PanTT26 TILs.

**Fig. 2:**
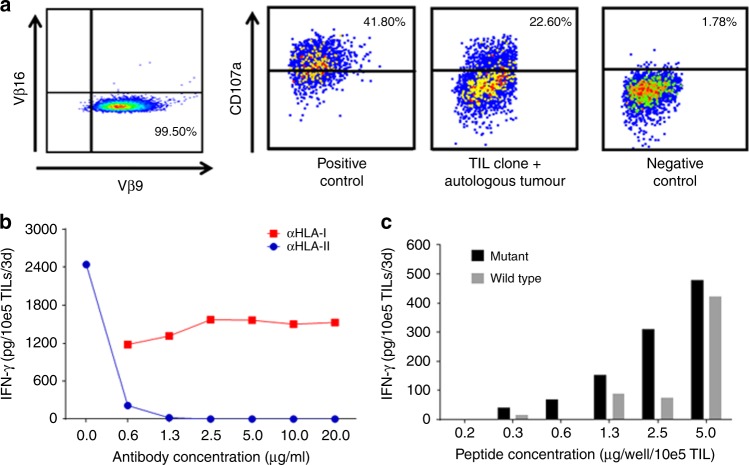
Characterisation of a specific CD4^+^ TIL clone from patient PanTT39. **a** The CD4^+^ TIL clone obtained from patient PanTT39 after IL-2, IL-15 and IL-21 stimulation stained for TCR Vβ9. After 5 h of incubation with the autologous tumour cell line, the CD4^+^ TIL clone (directed against GLLR**Y**WRTERLF) from patient PanTT39 was analysed by flow cytometry for induction of surface CD107a expression. Compared to baseline, there was an approximate 20% increase in cytotoxic activity against the autologous tumour cell line, indicating that this CD4^+^ TIL clone possesses anti-tumour activity characterised by IFN-γ production as well as cytotoxicity. **b** The CD4^+^ TIL clone was co-cultured with the K7N7A8-derived peptide GLLR**Y**WRTERLF either in the presence of the anti-HLA class I antibody (clone W6/32) or the anti-HLA class II antibody (clone L243). Culture supernatants were collected 3 days later for IFN-γ measurement by ELISA. Blockade of the HLA class II antigen presentation showed the strongest antagonistic effect on IFN-γ production. **c** Dose-dependent activity of the CD4^+^ TIL clone was measured by titrating the GLLR**Y**WRTERLF peptide (and the corresponding wild-type peptide GLLR**D**WRTERLF). Targeted activity—based on peptide-driven IFN-γ production—was differentially modulated (more cytokine induction) at lower concentrations of the mutated peptide. A high concentration of peptides, ie, 5 μg peptide per well/10*e*5 TIL, resulted in similar IFNγ production to mutant and wild-type peptides. TCRs that recognise the mutant as well as wild-type epitopes are likely to produce a similar response; minor T-cell subpopulations with different TCRs that which recognise preferentially private mutated targets—can be singled out in culture when exposed to lower peptide concentrations (0.3–2.5 μg peptide/10*e*5 TIL)

PanTT39 TILs produced lower IFN-γ/10*e*5 TIL in response to mutated peptides (Table 2B) as compared to PanTT26 TILs. We considered the possibility that CD4^+^ T cells in PanTT39 TILs could comprise a mixture of different T-cell subsets, eg, Th1, Th2 and Th17. In order to better define TIL PanTT30 reactivity, we obtained a CD4^+^ T-cell clone from PanTT39 TILs by limiting dilution. Flow cytometric analysis revealed that the CD4^+^ TIL clone was TCR Vβ9^+^ (Fig. 2a). Next, we intended to ascertain whether the CD4^+^ TCR Vβ9 TIL clone obtained was able to recognise any of the mutated peptides tested earlier in the screening assay to gauge for anti-cancer peptide-specific reactivity. TILs were co-incubated with the same panel of HLA class II-binding peptides for 3 days, after which IFN-γ production in the supernatant was detected by ELISA. A single mutated peptide was strongly recognised by the CD4^+^ TIL clone, namely GLLR**Y**WRTERLF (wild-type sequence: GLLR**D**WRTERLF), which derives from an uncharacterised protein product of 449 amino acids encoded by the *K7N7A8* gene. The CD4^+^ TCR Vβ9^+^ TIL clone that recognises the K7N7A8 mutated peptide GLLR**Y**WRTERLF produced a cytotoxic T-cell response against the autologous tumour cell line, which was assessed in a standard CD107a induction assay (Fig. 2a). In addition, the CD4^+^ TIL clone produced 480 mg/ml IFN-γ in response to GLLR**Y**WRTERLF, compared to a meagre 6 pg IFN-γ/10*e*5 TIL by the ‘young’ mixed TILs. Reactivity of the CD4^+^ TIL clone to the mutated peptide GLLR**Y**WRTERLF could be blocked with the L243 antibody (anti-HLA class-II, DR) in a dose-dependent manner (Fig. 2b). No difference in peptide reactivity of the TIL clone was observed in the presence of the W6/32 antibody (anti-HLA-I), further affirming that GLLR**Y**WRTERLF contained a nominal HLA class II neoepitope. Using peptide titration, we observed that the GLLR**Y**WRTERLF mutated peptide induced robust IFN-γ production by the CD4^+^ Vβ9^+^ TIL clone from patient PanTT39 even at low peptide concentrations, indicating the presence of high-affinity TCRs (Fig. 2c). The wild-type peptide was also able to activate T cells at the high concentration of 5 μg peptide per well, since TCR signal strength is affected by antigen affinity as well as antigen dose.^26^ Online BLAST analysis (UniProt KB and NCBI protein database) revealed that the peptide belongs to a putative water channel transporter like aquaporin 1 (AQP1) (Supplementary Figure 3).

**Table 2B:** ▓

				IFN-γ (pg/10*e*5 TIL/1 μg peptide)	
Peptide ID	Wild-type sequence	Mutated sequence	Gene name	Wild type	Mutated
PanTT39-P1	AFTLLLYCELLQWED	AFTMLLYCELLQWED	*DOCK3*	29	28
PanTT39-P2	TIYSLFYSVADRDAPA	TIYSLFYSVADQDAPA	*AQP7*	21	1
PanTT39-P3	FKQDLMIEDNLL	FKQDLMLEDNLL	*CCDC39*	4	0
PanTT39-P4	QDLMIEDNLLKLEV	QDLMLEDNLLKLEV	*CCDC39*	0	11
PanTT39-P5	GLLRDWRTERLF	GLLRYWRTERLF	*K7N7A8*	0	6
PanTT39-P6	ILFSLQPGLLRDW	ILFSLQPGLLRYW	*K7N7A8*	21	6
PanTT39-P7	NVSFFHYPEYGY	NVSFFHYQEYGY	*TENM3*	16	17
PanTT39-P8	EFPVRQAAAIYLK	EFPVLQAAAIYLK	*IPO8*	14	10
PanTT39-P9	INFKIERGQLLAV	INFKIERGQLAV	*CFTR*	6	0
PanTT39-P10	GAIVIERPNVKWS	GAIVIELPNVKWS	*VPS4B*	6	2
PanTT39-P11	LNKVTIDARHRLPL	LNKVTIDAIHRLPL	*MORC1*	9	14
PanTT39-P12	DFGFARTLAAPGDI	DFGFALTLAAPGDI	*CDKL3*	11	6
PanTT39-P13	LELMNKLLSPVVPQ	LELINKLLSPVVPQ	*NUP93*	0	19
PanTT39-P14	IEELRHLWDLLLELTL	IEELHHLWDLLLELTL	*SPTA1*	16	0

Antigen-specific IFN-γ production to mutated and the corresponding wild-type target by ‘young’ TILs from patient PanTT39.

*WT* wild type, *Mut* mutant

**Fig. 3:**
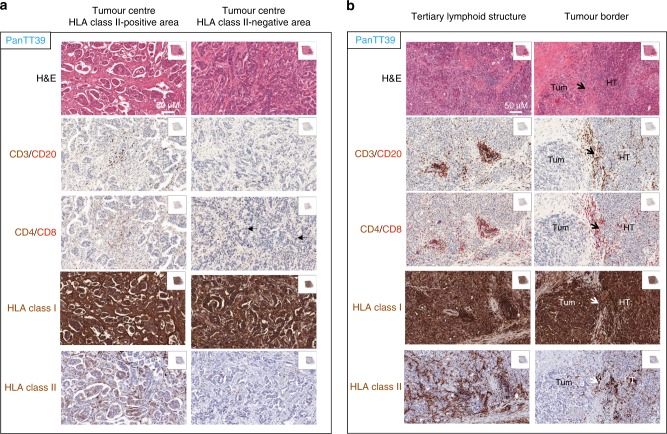
Immunostaining of tumour tissue from patient PanTT39. Four-micrometre sections of resected tumour tissue from patient PanTT39 was processed and used for immunohistochemistry as described for patient PanTT26. Expression of HLA class I and class II molecules in the tumour tissue was also performed. The black arrows in the tumour border immunostaining indicate the cellular rim that separates the diseased tissue and tumour mass from healthy pancreas parenchyma. **a** A closer look at immunostaining within the centre of the tumour mass, distinguished either by HLA class II-positive (left panels) or negative expression (right panels) revealed that CD4^+^ T cells almost exclusively infiltrated the HLA-class II-positive area although clusters of CD8^+^ TILs are also visible (indicated by dark ochre arrows, third panel on the right). Also, in the HLA class II-negative sections, it appears that sporadic expression of the MHC class II molecule can still be seen although to a much lesser extent. **b** Strong T-cell infiltration can be seen at the tumour border, which appears to be dominated by CD8^+^ TILs. Except for a small sub-region in the centre of the tumour mass, where the tumour cells stained weakly for HLA-class II molecule expression, no HLA class II expression was found to be associated with tumour cells. Tum tumour, HT healthy tissue

Akin to PanTT26, immunohistology of PanTT39 tumour showed weak T-cell infiltration into the centre of the tumour mass (Fig. 3a) compared to their accumulation at the tumour border as well as in the tertiary lymphoid structures (TLS) (Fig. 3b). Nevertheless, CD4^+^ T cells appeared to infiltrate into HLA class II-positive areas within the tumour centre (Fig. 3a). Interestingly, a similar profile was observed for B cells present at the tumour border, albeit at a much lower density than T cells. The cluster of CD20^+^ B cells appeared to be surrounded by T-cell populations—the majority of which are CD8^+^. This also holds true for CD8^+^ TILs that accumulate at the tumour border. The most striking difference between the lymphocytic infiltrate between the PDAC tissue from patients PanTT26 and PanTT39 is that TLS from PanTT26 are significantly larger with well-defined populations of mainly CD4^+^ T cells (as well as CD8^+^ T cells) surrounding B cells.

### Patient PanTT77

**Fig. 4:**
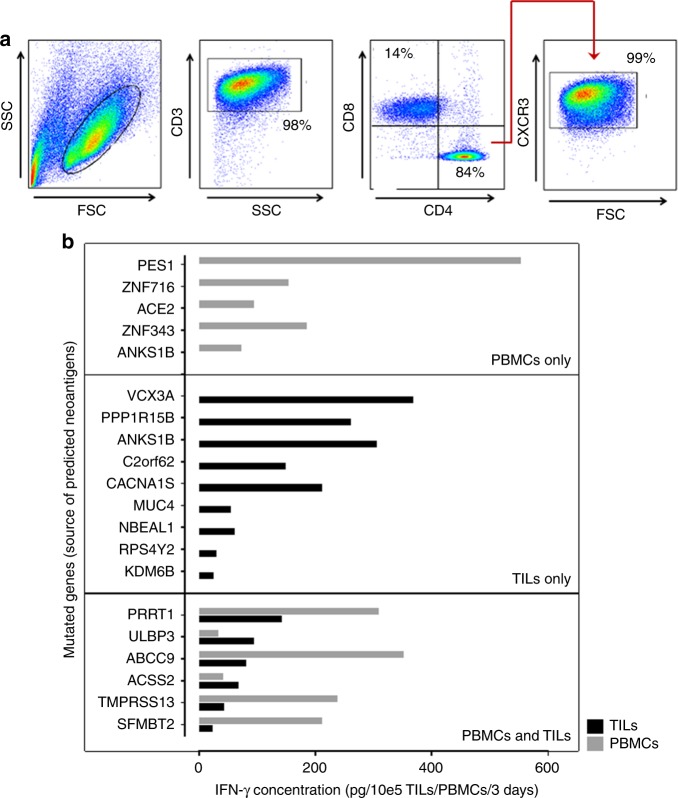
Characterisation of TILs from patient PanTT77. **a** TILs from patient PanTT77 were composed of almost 84% of CD4^+^ T cells and 14% of CD8^+^ T cells. CD4^+^ TILs from this patient were found to express the CXCR3 protein on their surface (98.8%). **b** Private mutated peptides generated based on whole-exome sequencing data of the tumour tissue from patient PanTT77 were co-incubated with the autologous PBMCs or TILs for 3 days, after which IFN-γ production in the culture supernatants was measured by ELISA. The PBMCs were found to respond to five mutated peptides while TILs reacted to nine mutated peptides. However, six mutated peptides elicited T-cell reactivity in PBMCs as well as in TILs

PanTT77 TILs comprised ~84% CD4^+^ T cells and 14% CD8^+^ T cells (Fig. 4a). Immunoreactivity of PBMCs as well as TILs from this patient to a panel of mutant and wild-type peptide sequences was assessed. A tumour cell line could not be established due to limited tissue availability since TIL propagation and whole-exome sequencing was performed from a single 2 mm needle biopsy. A unique peptide recognition profile marked by IFN-γ production was observed in PBMCs (five mutated peptides) and in TILs (nine mutated peptides) (Fig. 4b), showing that PBMCs from patient PanTT77 had a rather broad recognition of private neoepitopes without in vitro re-stimulation. A set of mutant peptides were only recognised by TILs, eg, the Protein Phosphatase 1 Regulatory Subunit 15B (PPP1R15B), which is part of an enzyme that dephosphorylates the eukaryotic translation initiation factor 2A (involved in regulating RNA translation into proteins) in response to stress, and with pro-oncogenic characteristics in breast cancer;^27^ neurobeachin-like protein 1 (NBEAL1), a protein that is expressed in the brain, testes and kidneys but overexpressed in gliomas;^28^ Ankyrin Repeat And Sterile Alpha Motif Domain Containing 1B (ANKS1B), which is expressed in normal brain tissue and is required for development, but also implicated in the pathogenesis of Alzheimer’s Disease and downregulated in smoking-related clear-cell renal cell carcinoma;^29,30^ Ciliogenesis Associated TTC17 Interacting Protein (CATIP/C2orf62), a protein involved in cilium biogenesis by inducing actin polymerisation;^31^ Calcium Voltage-Gated Channel Subunit Alpha1 S (CACNA1S), a subunit of a voltage-gated calcium channel with an important role in interacting with the ryanodine receptor in muscle cells for excitation–contraction coupling.^32^ Interestingly, some of the mutated peptides were recognised by TILs and PBMCs (six mutated peptides) and triggered stronger IFN-γ production in PBMCs (up to IFN-γ 350 pg/10*e*5 PBMCs) compared to TILs (up to 141 pg IFN-γ/10*e*5 TIL). A single mutated peptide, derived from the Proline Rich Transmembrane Protein 1 (PRRT1, also known as SynDIG4), induced strong IFN-γ by PBMCs and TILs. PRRT1 is part of the α-amino-3-hydroxy-5-methyl-4-isoxazolepropionic acid receptor (AMPAR) complex, which is involved in glutamate transport in the central nervous system and is important for synaptic transmission.^33,34^ No mutations were found in the HLA class I and class II pathways in this patient’s tumour.

## Discussion

We have been able to reliably grow TILs from patients with pancreatic cancer that could be used for the adoptive cellular therapy. Using a combination of co-culture techniques, we gauged the capacity, breadth and specificity of an individual patient’s T cells to react to private neoepitopes. To the best of our knowledge, this is the first report to describe individual neoepitope recognition patterns in patients with pancreatic cancer and argues that pancreatic cancer exhibits a certain degree of ‘immunogenicity’, making this malignancy potentially amenable to immunotherapeutic approaches. An integral component of the present study is the use of autologous tumour cell lines to profile TIL neoepitope recognition. The availability of samples for this purpose can be scarce, since surgery is mostly performed for patients who present without metastasis at diagnosis. Thus, the use of PBMCs to screen for neoepitope recognition is also a viable approach for developing personalised cellular therapies.

Repeated exposure to particular antigenic targets is likely to enrich for certain T-cell populations capable of durable anti-tumour responses. This is based on the differential recognition of mutated peptides (as opposed to the wild-type/native form), arising from important driver mutations. This has been shown for the KRAS_G12D_ mutation in a patient with metastatic colorectal cancer,^15^ and we observed that repetitive exposure to autologous tumour cells can result in enrichment of KRAS-specific T cells that was non-detectable in the ‘young’ TILs population.

TILs derived from patient PanTT39 comprised mainly CD4^+^ T cells. The IFN-γ-producing capacity of CD4^+^ TILs directed against cancer neoepitopes may confer productive clinical responses, as shown in a patient with cholangiocarcinoma (directed against mutated ERBB2IP).^13^ Several other CD4^+^ T-cell neoantigens have been described and concisely reviewed elsewhere,^35^ a majority of which are restricted by the HLA-DR alleles. Of note, mutations within the HLA class I alleles, mostly in HLA-A and HLA-C (Supplementary Table 2), were identified in the tumour lesion obtained from patient PanTT39, which may have given rise to preferential expansion of CD4^+^ T cells recognising the nominal tumour target antigen bound to HLA class II molecules. This underlines the fact that tumour immune-escape, associated with the HLA class I or class II antigen processing and presentation machinery needs to be implemented in the planning of immunotherapeutic strategies. Mutations in the HLA class I or class II molecules could therefore exclude an entire set of neoepitopes that would otherwise be visible to CD8^+^ T cells, thus facilitating tumour escape.^36,37^ However, tumour-infiltrating antigen-presenting cells (APCs), professional and non-professional alike, including CD8^+^ and gamma-delta (γδ) T cells that express HLA-DR^38,39^ may present tumour-associated antigens to CD4^+^ TILs in the tumour microenvironment, even when the tumour itself may harbour mutations in the HLA class II pathway. Expanded TILs from patient PanTT77 were also enriched for CD4^+^ T cells coupled with broad neoepitope recognition despite the absence of mutations in either HLA pathway, thus underlining the importance of CD4^+^ T cells in targeted immunotherapy.

Furthermore, four mutations were found in HLA class II molecules in patient PanTT39 (two mutations in HLA-DRB1 and two mutations in HLA-DPA1). However, the antigen processing and presentation machinery was not compromised, as reflected by the strong (and almost exclusive) CD4^+^ TIL response. Interestingly, the immunostaining results showed a dominant composition of CD4^+^ TILs in the lymphocytic infiltrate in tumour sections from patient PanTT39 (including the TLS). Considering that this patient harboured mutations in the HLA-I pathway, the CD4^+^ TILs were likely to be the ‘fitter’ T-cell subpopulation that managed to expand in vitro in response to IL-2, IL-15 and IL-21 stimulation. In contrast, patient PanTT26 did not have any mutations in the MHC-I pathway (like patient PanTT77) and displayed a prominent T-cell presence especially in the TLS. IL-2, IL-15 and IL-21 conditioning of total TILs from the resected PanTT26 tumour piece (which includes the TLS) was enriched for CD8^+^ T cells, as confirmed in the cytotoxicity assay performed with the autologous tumour cell line. The same TILs, following 3× stimulation with the autologous tumour cell line, yielded a CD8^+^ T-cell population, which included a KRAS_G12V_ reactive subset. Previous study showed large lymphocytic infiltration in the tumour-associated TLS (also referred to as tertiary lymphoid organs, TLO) may reflect survival benefit in patients with solid tumours including pancreatic cancer.^40,41^ The survival period post-tissue sampling recorded for patient PanTT26 (547 days) was much longer than for patient PanTT39 (411 days) (Table 1). TILs that are isolated from TLS/TLO may, in fact, represent ‘healthier’ and functionally superior effector cells with the capacity to impede tumour growth under amenable conditions, ie, with cytokine supplementation. A ‘precision’ TIL harvest of the future would, therefore, not rely solely on stochastic tumour sections, yet possibly on micro-dissected TLS/TLO regions.

The PanTT39 CD4^+^ TIL clone recognised a mutant peptide derived from the aquaporin (AQP)-like putative transmembrane ion/water channel K7N7A8 protein product, which also induced potent cytotoxicity against the autologous tumour cell line. At least 40 different mutations in the *AQP2* gene (resulting in protein product variants) have been linked to the onset of autosomal nephrogenic diabetes insipidus, where loss of responsiveness to vasopressin disrupts the kidneys’ ability to concentrate urine, resulting in an excessive excretory volume.^42^ Although site-specific mutations in the AQP4 molecule have been associated with reduced binding of potentially pathogenic IgG (in conjunction with the neurodegenerative disease neuromyelitis optica),^43^ clinically relevant immune responses to mutant AQPs remain unknown. To the best of our knowledge, the present study is the first to describe CD4^+^ TIL anti-tumour responses in pancreatic cancer directed against a mutant epitope derived from an AQP-like molecule.

Of note, specific immunomodulatory processes linked to the pancreas would inevitably affect the outcome of TIL therapy. For instance, stimulation of the vagus nerve serving the pancreas is likely to induce insulin production while inhibiting hepatic glucose release.^44^ Vagus nerve stimulation also reduces tumour necrosis factor-α production by gut macrophages.^45^ Intratumoural TGF-β, associated with disease progression, is a known suppressor of effective anti-tumour immune responses in pancreatic cancer.^46^ Strong TGF-β production in the tumour microenvironment impedes TIL activity and reduces the chances of tumour regression.^47,48^ Low-dose gemcitabine as well as cyclophosphamide can lead to reduction in regulatory T-cell (Treg) numbers, a major source of TGF-β and represents an effective strategy to improve local anti-tumour immune responses.^49–51^ Cyclophosphamide is already used as a pre-emptive drug in adoptive cell therapy clinical protocols.^52^ Interestingly, pharmacological depletion of Tregs using cyclophosphamide results in the reduction of intracellular adenosine triphosphate (ATP) turnover^53^ and their impaired ability to repair DNA double-strand breaks (DSBs).^51^ Surface-bound CD73 is an ecto-5′-nucleotidase that dephosphorylates AMP to produce adenosine, which goes on to trigger several different pathways in the tumour microenvironment including but not limited to aberrant neovascularisation, conversion of inflammatory M1 macrophages to anti-inflammatory M2 macrophages and apoptosis of tumour-infiltrating T cells.^54^ Hypoxia and an overt inflammatory milieu can lead to CD73 upregulation on stromal and tumour cells, which would then create a rather inhospitable environment  for tumour-directed immune cells. Anti-CD73 strategies are now in clinical assessment as an adjunctive immunotherapeutic measure to complement anti-PD-1/PD-L1 therapy for cancer, qualifying CD73 as a novel immune checkpoint (ClinicalTrials.gov identifiers: NCT03454451; NCT03381274; NCT03549000). Increased potassium channel activity and its dysregulation is yet another characteristic of solid tumours linked to invasiveness and uncontrolled cancer cell proliferation.^55^ At least three different potassium channel genes are known to be overexpressed in pancreatic cancer. Immunologically, dying tumour cells release an excess of potassium into the tumour microenvironment, which has a deleterious effect on infiltrating T cells by impairing the TCR-Akt-mTOR signalling cascade for effector functions, including IFN-γ production.^56,57^ Thus, the effect of induction of cell death as well as specific immune-suppressive activity in the tumour microenvironment and how they influence adoptive T-cell therapies warrant consideration in developing cellular therapies.

Satellite studies performed alongside clinical trials of novel cancer immunotherapies, ie, vaccines, immune checkpoint blockade or TIL therapy show that immune correlates of protection (corresponding to immune-related response evaluation criteria in solid tumours (irRECIST)) observed in responding patients are partly concomitant with the emergence of specific populations of neoantigen-directed T cells in peripheral blood.^12,58–69^ Exposure to antigens, either by direct contact with tumour cells or associated products presented by professional APCs or exosomes^70^, may activate T cells bearing TCRs that are either silenced or tolerised by the tumour.

Needle biopsies (such as for patient PanTT77) can also be used to cultivate TILs. Alternatively, biopsy material may also be used for constructing the patient’s mutanome to help screen for neoepitope-reactive circulating T cells, particularly if a limited number of tumour mutations are targeted by a specific TIL product. Future studies will also focus on whether gamma-delta (γδ) TCRs can recognise mutated targets relevant to cancer immunotherapy, although we did not observe TCR γδ+populations in the three TIL lines examined in greater detail in this report.

Several T-cell-based approaches to treat pancreatic cancer are currently pursued. The NCI is currently carrying out a clinical study of IL-2-stimulated TIL infusion in patients with metastatic pancreatic cancer (ClinicalTrials.gov identifier: NCT01174121). Another study is evaluating the safety and efficacy of EGFR-directed bispecific antibody-expressing T cells (BATs) in patients with locally advanced or metastatic pancreatic cancer who have already undergone one to two rounds of chemotherapy (ClinicalTrials.gov identifier: NCT03269526), although the T cells themselves will be harvested from blood. IL-2, IL-15 and IL-21-stimulated TIL therapy is actively pursued at the Krankenhaus Nordwest (KHNW, Frankfurt, Germany) for patients with resectable locally advanced or metastatic pancreatic cancer. Future clinical studies are likely to benefit from translational data linking anti-tumour T cells in pancreatic cancer to recognition of specific private mutations to improve survival as it was recently shown for a patient with metastatic breast cancer.^71^

## Conclusion

The results presented in this study have clinically relevant implications: (i) CD4^+^ TILs with TCRs directed against neoepitopes may kill autologous tumour cells and (ii) repeated exposure of TILs to the tumour cells (expressing neoantigens) can refine the TCR repertoire to produce a more focused immune response; (iii) PBMCs may be used as a viable source for T cells directed against tumour mutations and (iv) microdissection of tumour tissue may help achieve a more precise extraction and biologically relevant expansion of TILs.

## Electronic supplementary material

Supplementary Tables S1-S3

Supplementary Figure S1

Supplementary Figure S2

Supplementary Figure S3

## References

[1] WHO. *World Cancer Report 2014* (International Agency for Research on Cancer, World Health Organisation, Lyon, 2014).

[2] Benson, A., Olsen, J. & Sasson, A. In *Cancer Management: A Multidisciplinary Approach: Cancer Network* (eds Haller, D., Wagman, L., Camphausen, K. & Hoskins, W), (UBM Medica LLC Cancer Network, Online resource) (2016).

[3] Von Hoff DD (2011). Gemcitabine plus nab-paclitaxel is an active regimen in patients with advanced pancreatic cancer: a phase I/II trial. J. Clin. Oncol..

[4] Von Hoff DD (2013). Increased survival in pancreatic cancer with nab-paclitaxel plus gemcitabine. N. Engl. J. Med..

[5] Conroy T (2011). FOLFIRINOX versus gemcitabine for metastatic pancreatic cancer. N. Engl. J. Med..

[6] Gourgou-Bourgade S (2013). Impact of FOLFIRINOX compared with gemcitabine on quality of life in patients with metastatic pancreatic cancer: results from the PRODIGE 4/ACCORD 11 randomized trial. J. Clin. Oncol..

[7] Cao H, Le D, Yang LX (2013). Current status in chemotherapy for advanced pancreatic adenocarcinoma. Anticancer Res..

[8] Rosenberg SA (1985). Observations on the systemic administration of autologous lymphokine-activated killer cells and recombinant interleukin-2 to patients with metastatic cancer. N. Engl. J. Med..

[9] Lotze MT (1986). High-dose recombinant interleukin 2 in the treatment of patients with disseminated cancer. Responses, treatment-related morbidity, and histologic findings. JAMA.

[10] Topalian SL, Rosenberg SA (1987). Therapy of cancer using the adoptive transfer of activated killer cells and interleukin-2. Acta Haematol..

[11] Rosenberg SA (1988). Use of tumor-infiltrating lymphocytes and interleukin-2 in the immunotherapy of patients with metastatic melanoma. A preliminary report. N. Engl. J. Med..

[12] Gubin, M. M., Artyomov, M. N., Mardis, E. R. & Schreiber, R. D. Tumor neoantigens: building a framework for personalized cancer immunotherapy. *J. Clin. Invest.* **1125**, 3413–3421 (2015). 10.1172/JCI80008 PMC4588307 26258412

[13] Tran E (2014). Cancer immunotherapy based on mutation-specific CD4^+^ T cells in a patient with epithelial cancer. Science.

[14] Tran E (2015). Immunogenicity of somatic mutations in human gastrointestinal cancers. Science.

[15] Tran E (2016). T-cell transfer therapy targeting mutant KRAS in cancer. N. Engl. J. Med..

[16] Meng Q (2016). Expansion of tumor-reactive T cells from patients with pancreatic cancer. J. Immunother..

[17] Liu, Z. et al. Tumor-infiltrating T-cells (TIL) from patients with glioma. *Oncoimmunology* **6**, e1252894 (2016). 10.1080/2162402X.2016.1252894 PMC5353900 28344863

[18] Jones S (2010). Frequent mutations of chromatin remodeling gene *ARID1A* in ovarian clear cell carcinoma. Science.

[19] Eser S, Schnieke A, Schneider G, Saur D (2014). Oncogenic KRAS signalling in pancreatic cancer. Br. J. Cancer.

[20] Garcia-Silva S, Aranda A (2004). The thyroid hormone receptor is a suppressor of ras-mediated transcription, proliferation, and transformation. Mol. Cell. Biol..

[21] Martinez-Iglesias OA (2016). Autoregulatory loop of nuclear corepressor 1 expression controls invasion, tumor growth, and metastasis. Proc. Natl Acad. Sci. USA.

[22] Yang W (2010). Genome-wide association study in Asian populations identifies variants in ETS1 and WDFY4 associated with systemic lupus erythematosus. PLoS Genet..

[23] Mayer-Barber KD (2014). Host-directed therapy of tuberculosis based on interleukin-1 and type I interferon crosstalk. Nature.

[24] Cassandri M (2017). Zinc-finger proteins in health and disease. Cell Death Discov..

[25] Waddell N (2015). Whole genomes redefine the mutational landscape of pancreatic cancer. Nature.

[26] Keck S (2014). Antigen affinity and antigen dose exert distinct influences on CD4 T-cell differentiation. Proc. Natl Acad. Sci. USA.

[27] Shahmoradgoli M (2013). Protein phosphatase 1, regulatory subunit 15B is a survival factor for ERα-positive breast cancer. Int J. Cancer.

[28] Chen J (2004). Identification and characterization of NBEAL1, a novel human neurobeachin-like 1 protein gene from fetal brain, which is upregulated in glioma. Brain Res. Mol. Brain Res..

[29] Eckel-Passow JE (2014). ANKS1B is a smoking-related molecular alteration in clear cell renal cell carcinoma. BMC Urol..

[30] Ghersi E, Vito P, Lopez P, Abdallah M, D’Adamio L (2004). The intracellular localization of amyloid beta protein precursor (AbetaPP) intracellular domain associated protein-1 (AIDA-1) is regulated by AbetaPP and alternative splicing. J. Alzheimer’s Dis..

[31] Bontems F (2014). C2orf62 and TTC17 are involved in actin organization and ciliogenesis in zebrafish and human. PLoS ONE.

[32] Wu J (2015). Structure of the voltage-gated calcium channel Cav1.1 complex. Science.

[33] von Engelhardt J (2010). CKAMP44: a brain-specific protein attenuating short-term synaptic plasticity in the dentate gyrus. Science.

[34] Kirk LM (2016). Distribution of the SynDIG4/proline-rich transmembrane protein 1 in rat brain. J. Comp. Neurol..

[35] Sun Z, Chen F, Meng F, Wei J, Liu B (2017). MHC class II restricted neoantigen: a promising target in tumor immunotherapy. Cancer Lett..

[36] Maeurer MJ (1996). Tumor escape from immune recognition: lethal recurrent melanoma in a patient associated with downregulation of the peptide transporter protein TAP-1 and loss of expression of the immunodominant MART-1/Melan-A antigen. J. Clin. Invest..

[37] Concha-Benavente F, Srivastava R, Ferrone S, Ferris RL (2016). Immunological and clinical significance of HLA class I antigen processing machinery component defects in malignant cells. Oral Oncol..

[38] Speiser DE (2001). Human CD8(+) T cells expressing HLA-DR and CD28 show telomerase activity and are distinct from cytolytic effector T cells. Eur. J. Immunol..

[39] Brandes M, Willimann K, Moser B (2005). Professional antigen-presentation function by human gammadelta T cells. Science.

[40] Sautes-Fridman C (2016). Tertiary lymphoid structures in cancers: prognostic value, regulation, and manipulation for therapeutic intervention. Front. Immunol..

[41] Hiraoka N (2015). Intratumoral tertiary lymphoid organ is a favourable prognosticator in patients with pancreatic cancer. Br. J. Cancer.

[42] Loonen AJ, Knoers NV, van Os CH, Deen PM (2008). Aquaporin 2 mutations in nephrogenic diabetes insipidus. Semin. Nephrol..

[43] Tuller F (2016). Characterization of the binding pattern of human aquaporin-4 autoantibodies in patients with neuromyelitis optica spectrum disorders. J. Neuroinflamm..

[44] Meyers EE, Kronemberger A, Lira V, Rahmouni K, Stauss HM (2016). Contrasting effects of afferent and efferent vagal nerve stimulation on insulin secretion and blood glucose regulation. Physiol. Rep..

[45] Bonaz B, Sinniger V, Pellissier S (2017). The vagus nerve in the neuro-immune axis: implications in the pathology of the gastrointestinal tract. Front. Immunol..

[46] Shen, W. et al. TGF-β in pancreatic cancer initiation and progression: two sides of the same coin. *Cell Biosci*. **7**, 39 (2017). 10.1186/s13578-017-0168-0 PMC5545849 28794854

[47] Thomas DA, Massague J (2005). TGF-beta directly targets cytotoxic T cell functions during tumor evasion of immune surveillance. Cancer Cell.

[48] Principe DR (2016). TGFβ signaling in the pancreatic tumor microenvironment promotes fibrosis and immune evasion to facilitate tumorigenesis. Cancer Res..

[49] Shevchenko I (2013). Low-dose gemcitabine depletes regulatory T cells and improves survival in the orthotopic Panc02 model of pancreatic cancer. Int. J. Cancer.

[50] Sistigu A (2011). Immunomodulatory effects of cyclophosphamide and implementations for vaccine design. Semin. Immunopathol..

[51] Heylmann D (2013). Human CD4^+^CD25^+^ regulatory T cells are sensitive to low dose cyclophosphamide: implications for the immune response. PLoS ONE.

[52] Rosenberg SA, Restifo NP (2015). Adoptive cell transfer as personalized immunotherapy for human cancer. Science.

[53] Zhao J (2010). Selective depletion of CD4^+^CD25^+^Foxp3^+^ regulatory T cells by low-dose cyclophosphamide is explained by reduced intracellular ATP levels. Cancer Res..

[54] Antonioli L, Yegutkin GG, Pacher P, Blandizzi C, Haskó G (2016). Anti-CD73 in cancer immunotherapy: awakening new opportunities. Trends Cancer.

[55] Huang X, Jan LY (2014). Targeting potassium channels in cancer. J. Cell Biol..

[56] Eil RL (2015). Elevated potassium levels suppress T cell activation within tumors. J. Immunother. Cancer.

[57] Eil R (2016). Ionic immune suppression within the tumour microenvironment limits T cell effector function. Nature.

[58] McGranahan N (2016). Clonal neoantigens elicit T cell immunoreactivity and sensitivity to immune checkpoint blockade. Science.

[59] Valdez H (2000). Response to immunization with recall and neoantigens after prolonged administration of an HIV-1 protease inhibitor-containing regimen. ACTG 375 team. AIDS Clinical Trials Group. Aids.

[60] Gros A (2014). PD-1 identifies the patient-specific CD8(^+^) tumor-reactive repertoire infiltrating human tumors. J. Clin. Invest..

[61] Carreno BM (2015). Cancer immunotherapy. A dendritic cell vaccine increases the breadth and diversity of melanoma neoantigen-specific T cells. Science.

[62] Cohen CJ (2015). Isolation of neoantigen-specific T cells from tumor and peripheral lymphocytes. J. Clin. Invest..

[63] Kowalewski DJ, Stevanovic S, Rammensee HG, Stickel JS (2015). Antileukemia T-cell responses in CLL - we don’t need no aberration. Oncoimmunology.

[64] Rizvi NA (2015). Cancer immunology. Mutational landscape determines sensitivity to PD-1 blockade in non-small cell lung cancer. Science.

[65] Schumacher TN, Schreiber RD (2015). Neoantigens in cancer immunotherapy. Science.

[66] Gros A (2016). Prospective identification of neoantigen-specific lymphocytes in the peripheral blood of melanoma patients. Nat. Med..

[67] Lu YC, Robbins PF (2016). Cancer immunotherapy targeting neoantigens. Semin. Immunol..

[68] Lu YC, Robbins PF (2016). Targeting neoantigens for cancer immunotherapy. Int. Immunol..

[69] Tureci O (2016). Targeting the heterogeneity of cancer with individualized neoepitope vaccines. Clin. Cancer Res..

[70] Greening DW, Gopal SK, Xu R, Simpson RJ, Chen W (2015). Exosomes and their roles in immune regulation and cancer. Semin. Cell Dev. Biol..

[71] Zacharakis N (2018). Immune recognition of somatic mutations leading to complete durable regression in metastatic breast cancer. Nat. Med..

