# Supplementary material for: Neoepitope targets of tumour-infiltrating lymphocytes from patients with pancreatic cancer
Source: Br J Cancer. 2018 Oct 31;120(1):97–108. doi: 10.1038/s41416-018-0262-z (PMC6325142; doi:10.1038/s41416-018-0262-z)
Supplement: Supplementary file 1 — Supplementary Tables S1-S3 [file 41416_2018_262_MOESM1_ESM.doc]

**Supplementary Table 1**

|  |  |  |  | IFN-γ (pg/10^5^ TILs/1μg peptide) | | | |
| --- | --- | --- | --- | --- | --- | --- | --- |
|  |  |  |  | Young TIL | | Post-3X stim. with ATCL | |
| Peptide ID | Wildtype sequence | Mutated sequence | Gene Name | WT | Mut | WT | Mut |
| PanTT26-P1 | FEGTEMWNPNRELSE | FEGTEMWYPNRELSE | [ACHE](http://www.genecards.org/cgi-bin/carddisp.pl?gene=ACHE) | 104 | 14 | 228 | 0 |
| PanTT26-P2 | AVKRLPLVYCDYHGH | AVKRLPLIYCDYHGH | [AGTPBP1](http://www.genecards.org/cgi-bin/carddisp.pl?gene=AGTPBP1) | 60 | 149 | 0 | 0 |
| PanTT26-P3 | TCSCQSSGTSSTSYS | TCSCQSSWTSSTSYS | AK302451 | 30 | 0 | 0 | 0 |
| PanTT26-P4 | PWRKFPVYVLGQFLG | PWRKFPVHVLGQFLG | [AQP7](http://www.genecards.org/cgi-bin/carddisp.pl?gene=AQP7) | 152 | 315 | 0 | 443 |
| PanTT26-P5 | MNAAVTFANCALGRV | MNAAVTFTNCALGRV | [AQP7](http://www.genecards.org/cgi-bin/carddisp.pl?gene=AQP7) | 41 | 0 | 0 | 119 |
| PanTT26-P6 | INCLSSPNEETVLSA | INCLSSPSEETVLSA | [ARMC7](http://www.genecards.org/cgi-bin/carddisp.pl?gene=ARMC7) | 60 | 0 | 0 | 0 |
| PanTT26-P7 | STAYPAPMRRRCCLP | STAYPAPVRRRCCLP | [ARMC7](http://www.genecards.org/cgi-bin/carddisp.pl?gene=ARMC7) | 261 | 122 | 446 | 0 |
| PanTT26-P8 | VALKPQERVEKRQTP | VALKPQECVEKRQTP | [AUTS2](http://www.genecards.org/cgi-bin/carddisp.pl?gene=AUTS2) | 94 | 0 | 167 | 0 |
| PanTT26-P9 | PSHQPPASTLSPNPT | PSHQPPARTLSPNPT | [C5orf60](http://www.genecards.org/cgi-bin/carddisp.pl?gene=C5orf60) | 24 | 0 | 0 | 0 |
| PanTT26-P10 | TPEPAIPPKATLWPA | TPEPAIPHKATLWPA | [C6orf132](http://www.genecards.org/cgi-bin/carddisp.pl?gene=C6orf132) | 156 | 0 | 0 | 0 |
| PanTT26-P11 | MFTLTGCRLVEKT | MFTLTSCRLVEKT | [CCDC108](http://www.genecards.org/cgi-bin/carddisp.pl?gene=CCDC108) | 12 | 0 | 0 | 0 |
| PanTT26-P12 | THRPGGKHGRLAGGS | THRPGGKRGRLAGGS | [CCDC74B](http://www.genecards.org/cgi-bin/carddisp.pl?gene=CCDC74B) | 104 | 114 | 0 | 487 |
| PanTT26-P13 | VTVHPTSNSTATSQG | VTVHPTSKSTATSQG | [CD68](http://www.genecards.org/cgi-bin/carddisp.pl?gene=CD68) | 41 | 0 | 0 | 281 |
| PanTT26-P14 | STATHSPATTSHGNA | STATHSPSTTSHGNA | [CD68](http://www.genecards.org/cgi-bin/carddisp.pl?gene=CD68) | 36 | 0 | 0 | 0 |
| PanTT26-P15 | LQREYASVKEENERL | LQREYASMKEENERL | [CDK5RAP2](http://www.genecards.org/cgi-bin/carddisp.pl?gene=CDK5RAP2) | 77 | 0 | 0 | 0 |
| PanTT26-P16 | MEVSGCPTPAGQS | MEVSGWPTPAGQS | [CEACAM18](http://www.genecards.org/cgi-bin/carddisp.pl?gene=CEACAM18) | 75 | 0 | 98 | 0 |
| PanTT26-P17 | ARAAAAAAFEIDPRS | ARAAAAATFEIDPRS | [CELSR3](http://www.genecards.org/cgi-bin/carddisp.pl?gene=CELSR3) | 17 | 0 | 0 | 0 |
| PanTT26-P18 | HGLSHSLRQISSQLS | HGLSHSLWQISSQLS | [CEP164](http://www.genecards.org/cgi-bin/carddisp.pl?gene=CEP164) | 49 | 15 | 0 | 0 |
| PanTT26-P19 | VTTKKTPPSQPPGNV | VTTKKTPSSQPPGNV | [CNTN3](http://www.genecards.org/cgi-bin/carddisp.pl?gene=CNTN3) | 17 | 0 | 0 | 0 |
| PanTT26-P20 | SSLPGPPGPPGPPGP | SSLPGPPGPPGPRGY | [COL18A1](http://www.genecards.org/cgi-bin/carddisp.pl?gene=COL18A1) | 4 | 1 | 0 | 169 |
| PanTT26-P21 | FSISQLQKNHDMNDE | FSISQLQTNHDMNDE | [CYP7B1](http://www.genecards.org/cgi-bin/carddisp.pl?gene=CYP7B1) | 78 | 22 | 0 | 149 |
| PanTT26-P22 | GINQTTGALYLRVDS | GINQTTGTLYLRVDS | [DCHS1](http://www.genecards.org/cgi-bin/carddisp.pl?gene=DCHS1) | 12 | 0 | 0 | 0 |
| PanTT26-P23 | MSYDYHQNWGRDGG | MSYDYHHNWGRDGG | [DHX36](http://www.genecards.org/cgi-bin/carddisp.pl?gene=DHX36) | 110 | 46 | 164 | 0 |
| PanTT26-P24 | EMQEERLKLPILSEE | EMQEERLTLPILSEE | [DHX37](http://www.genecards.org/cgi-bin/carddisp.pl?gene=DHX37) | 70 | 0 | 41 | 0 |
| PanTT26-P25 | ENKNQELRSLISQYQ | ENKNQELHSLISQYQ | [DOCK3](http://www.genecards.org/cgi-bin/carddisp.pl?gene=DOCK3) | 8 | 0 | 0 | 0 |
| PanTT26-P26 | SSAEPTEHGERTPLA | SSAEPTENGERTPLA | [DPCR1](http://www.genecards.org/cgi-bin/carddisp.pl?gene=DPCR1) | 10 | 0 | 0 | 0 |
| PanTT26-P27 | VLACGLSRIWGEERG | VLACGLSQIWGEERG | [EDNRB](http://www.genecards.org/cgi-bin/carddisp.pl?gene=EDNRB) | 66 | 0 | 0 | 0 |
| PanTT26-P28 | LADGEGGGTDEGIYD | LADGEGGATDEGIYD | [EFS](http://www.genecards.org/cgi-bin/carddisp.pl?gene=EFS) | 20 | 190 | 0 | 0 |
| PanTT26-P29 | YVVPPPARPCPTSGP | YVVPPPAWPCPTSGP | [EFS](http://www.genecards.org/cgi-bin/carddisp.pl?gene=EFS) | 47 | 0 | 0 | 0 |
| PanTT26-P30 | MRETSGFTL | MRDKWLHIE | [PRSS3](http://www.genecards.org/cgi-bin/carddisp.pl?gene=PRSS3) | 77 | 0 | 0 | 0 |
| PanTT26-P31 | ECSECGKVFLESAAL | ECSECGKDFLESAAL | [ZNF264](http://www.genecards.org/cgi-bin/carddisp.pl?gene=ZNF264) | 81 | 0 | 0 | 0 |
| PanTT26-P32 | LTDHRAHRCPGDGDD | LTDHRAHCCPGDGDD | [ZNF423](http://www.genecards.org/cgi-bin/carddisp.pl?gene=ZNF423) | 45 | 0 | 0 | 0 |
| PanTT26-P33 | LTDHRAHRCPGGNAK | LTDHRAHCCPGGNAK | [ZNF423](http://www.genecards.org/cgi-bin/carddisp.pl?gene=ZNF423) | 72 | 18 | 0 | 0 |
| PanTT26-P34 | DEVSMKGRPPPTPLF | DEVSMKGGPPPTPLF | [FKBP15](http://www.genecards.org/cgi-bin/carddisp.pl?gene=FKBP15) | 63 | 0 | 0 | 0 |
| PanTT26-P35 | AQGWSTVARFQITAT | AQGWSTVSRFQITAT | [SLC25A23](http://www.genecards.org/cgi-bin/carddisp.pl?gene=SLC25A23) | 107 | 0 | 0 | 0 |
| PanTT26-P36 | KLVVVGAGGVGKSAL | KLVVVGAVGVGKSAL | [KRAS](http://www.genecards.org/cgi-bin/carddisp.pl?gene=KRAS) | 54 | 0 | 0 | 453 |
| PanTT26-P37 | LFGLGKDEGWGPPAR | LFGLGKDVGWGPPAR | [NT5C3B](http://www.genecards.org/cgi-bin/carddisp.pl?gene=NT5C3B) | 65 | 100 | 0 | 3 |
| PanTT26-P38 | VMMHGGPPHPGMPMS | VMMHGGPAHPGMPMS | [MEIS1](http://www.genecards.org/cgi-bin/carddisp.pl?gene=MEIS1) | 92 | 5 | 0 | 0 |
| PanTT26-P39 | MRHFCLISE | MHHFCLISE | [TMEM168](http://www.genecards.org/cgi-bin/carddisp.pl?gene=TMEM168) | 124 | 0 | 355 | 0 |
| PanTT26-P40 | PMEKPTISTEKPTIP | PMEKPTITTEKPTIP | [ZAN](http://www.genecards.org/cgi-bin/carddisp.pl?gene=ZAN) | 13 | 3 | 0 | 0 |
| PanTT26-P41 | YVSMMCNEQAYSLAV | YVSMMCNKQAYSLAV | [NDUFS2](http://www.genecards.org/cgi-bin/carddisp.pl?gene=NDUFS2) | 24 | 50 | 16 | 0 |
| PanTT26-P42 | LWTEGMLQMAFHILA | LWTEGMLKMAFHILA | [UBR1](http://www.genecards.org/cgi-bin/carddisp.pl?gene=UBR1) | 7 | 71 | 95 | 0 |
| PanTT26-P43 | KPVILGVRWYVETTS | KPVILGVCWYVETTS | [KLK6](http://www.genecards.org/cgi-bin/carddisp.pl?gene=KLK6) | 57 | 16 | 166 | 0 |
| PanTT26-P44 | TMLARLVSDS | TMLARLVLDS | [FAM161A](http://www.genecards.org/cgi-bin/carddisp.pl?gene=FAM161A) | 63 | 33 | 0 | 0 |
| PanTT26-P45 | SSGGGSSGGGYGGGS | SSGGGSSSGGYGGGS | [KRT10](http://www.genecards.org/cgi-bin/carddisp.pl?gene=KRT10) | 94 | 19 | 242 | 0 |
| PanTT26-P46 | DPSAIGLADPPIPSP | DPSAIGLVDPPIPSP | [SELV](http://www.genecards.org/cgi-bin/carddisp.pl?gene=SELV) | 114 | 20 | 37 | 0 |
| PanTT26-P47 | QLTAHKMIHTGEKPY | QLTAHKMNHTGEKPY | [ZNF100](http://www.genecards.org/cgi-bin/carddisp.pl?gene=ZNF100) | 46 | 45 | 0 | 0 |
| PanTT26-P48 | AVYTPPSVSTHQMPR | AVYTPPSDSTHQMPR | [PRSS3](http://www.genecards.org/cgi-bin/carddisp.pl?gene=PRSS3) | 22 | 17 | 0 | 0 |
| PanTT26-P49 | PGSGPQNPPGLGSGA | PGSGPQNAPGLGSGA | [LILRB3](http://www.genecards.org/cgi-bin/carddisp.pl?gene=LILRB3) | 0 | 0 | 0 | 0 |
| PanTT26-P50 | FASPGDDGDGRAEGF | FASPGDDRDGRAEGF | [MUC12](http://www.genecards.org/cgi-bin/carddisp.pl?gene=MUC12) | 9 | 12 | 0 | 0 |
| PanTT26-P51 | EPGDTALYLCASSQS | EPGDTALHLCASSQS | [TRBV23-1](http://www.genecards.org/cgi-bin/carddisp.pl?gene=TRBV23-1) | 6 | 37 | 0 | 0 |
| PanTT26-P52 | VNTTTSPVNTTTSPV | VNTTTSPANTTTSPV | [MS4A18](http://www.genecards.org/cgi-bin/carddisp.pl?gene=MS4A18) | 46 | 20 | 0 | 79 |
| PanTT26-P53 | GRKFAAWAPPSFSQT | GRKFAAWGPPSFSQT | [PTX4](http://www.genecards.org/cgi-bin/carddisp.pl?gene=PTX4) | 240 | 94 | 457 | 0 |
| PanTT26-P54 | EVPMCSDPEPRQEVP | EVPMCSDTEPRQEVP | [FAM120B](http://www.genecards.org/cgi-bin/carddisp.pl?gene=FAM120B) | 78 | 0 | 0 | 0 |
| PanTT26-P55 | KLSVAPSEVLEEDQV | KLSVAPSVVLEEDQV | [GGTA1P](http://www.genecards.org/cgi-bin/carddisp.pl?gene=GGTA1P) | 19 | 2 | 0 | 0 |
| PanTT26-P56 | DILEQARAAVDTYCR | DILEQARGAVDTYCR | [HLA-DRB1](http://www.genecards.org/cgi-bin/carddisp.pl?gene=HLA-DRB1) | 25 | 0 | 0 | 0 |
| PanTT26-P57 | TFNCHHARPWHNQFV | TFNCHHAQPWHNQFV | [HTR3D](http://www.genecards.org/cgi-bin/carddisp.pl?gene=HTR3D) | 60 | 34 | 0 | 0 |
| PanTT26-P58 | CVSMLGVPVDPDTLH | CVSMLGVLVDPDTLH | [HUWE1](http://www.genecards.org/cgi-bin/carddisp.pl?gene=HUWE1) | 0 | 5 | 0 | 0 |
| PanTT26-P59 | GYGEMGSGYREDLGA | GYGEMGSVYREDLGA | [IGFN1](http://www.genecards.org/cgi-bin/carddisp.pl?gene=IGFN1) | 21 | 39 | 0 | 488 |
| PanTT26-P60 | LLDRGSFRNDGLKAS | LLDRGSFWNDGLKAS | [KALRN](http://www.genecards.org/cgi-bin/carddisp.pl?gene=KALRN) | 42 | 87 | 0 | 354 |
| PanTT26-P61 | SQLMLTRKAEAALRK | SQLMLTRKGNASCLE | [KANSL1](http://www.genecards.org/cgi-bin/carddisp.pl?gene=KANSL1) | 196 | 0 | 74 | 0 |
| PanTT26-P62 | ALKIKGIHPYHSLSY | ALKIKGIRPYHSLSY | [KIAA1109](http://www.genecards.org/cgi-bin/carddisp.pl?gene=KIAA1109) | 177 | 139 | 65 | 0 |
| PanTT26-P63 | HNNIVYNEYISHREH | HNNIVYNKYISHREH | [KIN](http://www.genecards.org/cgi-bin/carddisp.pl?gene=KIN) | 0 | 5 | 0 | 0 |
| PanTT26-P64 | ARVILGVRWYVETTS | ARVILGVCWYVETTS | [KLK6](http://www.genecards.org/cgi-bin/carddisp.pl?gene=KLK6) | 40 | 28 | 0 | 62 |
| PanTT26-P65 | CQGDSGGPLVCGDHL | CQGDSGGLLVCGDHL | [KLK6](http://www.genecards.org/cgi-bin/carddisp.pl?gene=KLK6) | 27 | 0 | 0 | 0 |
| PanTT26-P66 | PVCSGASTSCCQQSS | PVCSGASSSCCQQSS | [KRTAP10-5](http://www.genecards.org/cgi-bin/carddisp.pl?gene=KRTAP10-5) | 0 | 7 | 0 | 0 |
| PanTT26-P67 | VPVAQVTTTSTTDAD | VPVAQVTMTSTTDAD | [KRTAP11-1](http://www.genecards.org/cgi-bin/carddisp.pl?gene=KRTAP11-1) | 25 | 44 | 0 | 103 |
| PanTT26-P68 | PRCCISSCCRPSCCV | PRCCISSFCRPSCCV | [KRTAP4-11](http://www.genecards.org/cgi-bin/carddisp.pl?gene=KRTAP4-11) | 56 | 189 | 0 | 325 |
| PanTT26-P69 | CRPQCCQSVCCQPTC | CRPQCCQTVCCQPTC | [KRTAP4-9](http://www.genecards.org/cgi-bin/carddisp.pl?gene=KRTAP4-9) | 101 | 0 | 279 | 0 |
| PanTT26-P70 | TCCRTTCYRPSCCVS | TCCRTTCFRPSCCVS | [KRTAP4-9](http://www.genecards.org/cgi-bin/carddisp.pl?gene=KRTAP4-9) | 217 | 118 | 67 | 0 |
| PanTT26-P71 | PGESLRPRGERRLPQ | PGESLRPLGERRLPQ | [LILRA6](http://www.genecards.org/cgi-bin/carddisp.pl?gene=LILRA6) | 37 | 0 | 0 | 0 |
| PanTT26-P72 | PGSGPQNRLGRYLEV | PGSGPQNGLGRYLEV | [LILRB3](http://www.genecards.org/cgi-bin/carddisp.pl?gene=LILRB3) | 111 | 0 | 0 | 0 |
| PanTT26-P73 | ETGPEAERLEQLESG | ETGPEAEWLEQLESG | [LOC642846](http://www.genecards.org/cgi-bin/carddisp.pl?gene=LOC642846) | 55 | 0 | 0 | 0 |
| PanTT26-P74 | QKEKSLEFTKELPGY | QKEKSLELTKELPGY | [LRRC37A3](http://www.genecards.org/cgi-bin/carddisp.pl?gene=LRRC37A3) | 64 | 0 | 0 | 0 |
| PanTT26-P75 | PFSPSHPAPPSDPSH | PFSPSHPGPPSDPSH | [MAGI2](http://www.genecards.org/cgi-bin/carddisp.pl?gene=MAGI2) | 90 | 24 | 0 | 0 |
| PanTT26-P76 | DEMDCPLSPTPPLCS | DEMDCPLRPTPPLCS | [MALRD1](http://www.genecards.org/cgi-bin/carddisp.pl?gene=MALRD1) | 117 | 51 | 222 | 527 |
| PanTT26-P77 | VKDQGPMVSAPVKDQ | VKDQGPMFSAPVKDQ | [MAP6](http://www.genecards.org/cgi-bin/carddisp.pl?gene=MAP6) | 136 | 0 | 0 | 0 |
| PanTT26-P78 | KDQGPIVPAPVKGEG | KDQGPIVTAPVKGEG | [MAP6](http://www.genecards.org/cgi-bin/carddisp.pl?gene=MAP6) | 4 | 0 | 0 | 0 |
| PanTT26-P79 | TTTASTEGSETTTAS | TTTASTECSETTTAS | [MUC22](http://www.genecards.org/cgi-bin/carddisp.pl?gene=MUC22) | 42 | 0 | 0 | 0 |
| PanTT26-P80 | LRPQLAENKQQFRNL | LRPQLAEKKQQFRNL | [NBPF1](http://www.genecards.org/cgi-bin/carddisp.pl?gene=NBPF1) | 75 | 125 | 0 | 0 |
| PanTT26-P81 | EKKQQFRNLKEKCFL | EKKQQFRSLKEKCFL | [NBPF10](http://www.genecards.org/cgi-bin/carddisp.pl?gene=NBPF10) | 169 | 212 | 0 | 164 |
| PanTT26-P82 | AFMYAKKEEWKKAEE | AFMYAKKGEWKKAEE | [NCF2](http://www.genecards.org/cgi-bin/carddisp.pl?gene=NCF2) | 29 | 60 | 0 | 0 |
| PanTT26-P83 | KLKKKQVNVFA | KLKKKQVKVFA | [NCOR1](http://www.genecards.org/cgi-bin/carddisp.pl?gene=NCOR1) | 106 | 327 | 0 | 710 |
| PanTT26-P84 | GRLILWEAPPLGAGG | GRLILWEGPPLGAGG | [NEK8](http://www.genecards.org/cgi-bin/carddisp.pl?gene=NEK8) | 97 | 32 | 0 | 164 |
| PanTT26-P85 | NQLKERSFAQLISKD | NQLKERSIAQLISKD | [NLRP14](http://www.genecards.org/cgi-bin/carddisp.pl?gene=NLRP14) | 108 | 0 | 108 | 0 |
| PanTT26-P86 | ILLIHCDAHLHTPMY | ILLIHCDTHLHTPMY | [OR2T4](http://www.genecards.org/cgi-bin/carddisp.pl?gene=OR2T4) | 61 | 35 | 0 | 0 |
| PanTT26-P87 | LLIHCDAHLHTPMYF | LLIHCDAYLHTPMYF | [OR2T4](http://www.genecards.org/cgi-bin/carddisp.pl?gene=OR2T4) | 74 | 43 | 0 | 0 |
| PanTT26-P88 | AVVFQDSVVFRVAPW | AVVFQDSMVFRVAPW | [PADI4](http://www.genecards.org/cgi-bin/carddisp.pl?gene=PADI4) | 19 | 21 | 0 | 0 |
| PanTT26-P89 | EHSQETESLREALLS | EHSQETEILREALLS | [PDE4DIP](http://www.genecards.org/cgi-bin/carddisp.pl?gene=PDE4DIP) | 9 | 15 | 0 | 0 |
| PanTT26-P90 | PYGCLPTGDRTGLIE | PYGCLPTRDRTGLIE | [PIK3CD](http://www.genecards.org/cgi-bin/carddisp.pl?gene=PIK3CD) | 9 | 0 | 0 | 0 |
| PanTT26-P91 | GLPTDTIRKEFRTRM | GLPTDTICKEFRTRM | [PLCB4](http://www.genecards.org/cgi-bin/carddisp.pl?gene=PLCB4) | 81 | 93 | 0 | 20 |
| PanTT26-P92 | TGAMNVAKGTIQTGV | TGAMNVAIGTIQTGV | [PLIN4](http://www.genecards.org/cgi-bin/carddisp.pl?gene=PLIN4) | 107 | 99 | 0 | 124 |
| PanTT26-P93 | TYSPTSPVYTPTSPK | TYSPTSPDYTPTSPK | [POLR2A](http://www.genecards.org/cgi-bin/carddisp.pl?gene=POLR2A) | 66 | 0 | 511 | 0 |
| PanTT26-P94 | CRGSGKSNVGTSGDH | CRGSGKSKVGTSGDH | [POTEH](http://www.genecards.org/cgi-bin/carddisp.pl?gene=POTEH) | 7 | 41 | 0 | 0 |
| PanTT26-P95 | SKMGKWCRHCFAWCR | SKMGKWCSHCFAWCR | [POTEH](http://www.genecards.org/cgi-bin/carddisp.pl?gene=POTEH) | 200 | 176 | 58 | 149 |
| PanTT26-P96 | SKMGKWCRHCFPCCR | SKMGKWCSHCFPCCR | [POTEJ](http://www.genecards.org/cgi-bin/carddisp.pl?gene=POTEJ) | 297 | 261 | 0 | 137 |
| PanTT26-P97 | GEPIPQPARLRYVTS | GEPIPQPVRLRYVTS | [PRICKLE2](http://www.genecards.org/cgi-bin/carddisp.pl?gene=PRICKLE2) | 12 | 27 | 0 | 0 |
| PanTT26-P98 | VQLRGRAQGGGALRA | VQLRGRALGGGALRA | [PRSS22](http://www.genecards.org/cgi-bin/carddisp.pl?gene=PRSS22) | 20 | 72 | 0 | 0 |
| PanTT26-P99 | NLVHGPPAPPQVGAD | NLVHGPPGPPQVGAD | [PSD](http://www.genecards.org/cgi-bin/carddisp.pl?gene=PSD) | 57 | 97 | 80 | 328 |
| PanTT26-P100 | GGGPDGPLYKVSVTA | GGGPDGPRYKVSVTA | [RAP1GAP](http://www.genecards.org/cgi-bin/carddisp.pl?gene=RAP1GAP) | 44 | 0 | 60 | 0 |
| PanTT26-P101 | GGHDSSSWSHRYGGG | GGHDSSSLSHRYGGG | [RBMXL3](http://www.genecards.org/cgi-bin/carddisp.pl?gene=RBMXL3) | 9 | 0 | 0 | 0 |
| PanTT26-P102 | VRRCLPLCALTLEAA | VRRCLPLWALTLEAA | [RHCE](http://www.genecards.org/cgi-bin/carddisp.pl?gene=RHCE) | 9 | 75 | 0 | 0 |
| PanTT26-P103 | PSRHRYGARQPRARL | PSRHRYGTRQPRARL | [RNF126](http://www.genecards.org/cgi-bin/carddisp.pl?gene=RNF126) | 152 | 233 | 0 | 142 |
| PanTT26-P104 | ETKTKDEMAAAEEKV | ETKTKDETAAAEEKV | [RNF213](http://www.genecards.org/cgi-bin/carddisp.pl?gene=RNF213) | 0 | 0 | 0 | 0 |
| PanTT26-P105 | QEVEGETQKTEGDAQ | QEVEGETHKTEGDAQ | [RP1L1](http://www.genecards.org/cgi-bin/carddisp.pl?gene=RP1L1) | 0 | 22 | 0 | 0 |
| PanTT26-P106 | KSEGEEAQEVEGETQ | KSEGEEAHEVEGETQ | [RP1L1](http://www.genecards.org/cgi-bin/carddisp.pl?gene=RP1L1) | 3 | 29 | 0 | 105 |
| PanTT26-P107 | AGRFGQGAHHAAGQA | AGRFGQGDHHAAGQA | [SBSN](http://www.genecards.org/cgi-bin/carddisp.pl?gene=SBSN) | 95 | 80 | 44 | 205 |
| PanTT26-P108 | QLLEGLGFTLTVVPE | QLLEGLGCTLTVVPE | [SERPINA13P](http://www.genecards.org/cgi-bin/carddisp.pl?gene=SERPINA13P) | 47 | 2 | 425 | 0 |
| PanTT26-P109 | TRLFPNEFANFYNAV | TRLFPNELANFYNAV | [SLITRK1](http://www.genecards.org/cgi-bin/carddisp.pl?gene=SLITRK1) | 47 | 46 | 0 | 0 |
| PanTT26-P110 | QQEIDQKRLEFEKQK | QQEIDQKKIRINAKT | [SMC1B](http://www.genecards.org/cgi-bin/carddisp.pl?gene=SMC1B) | 23 | 0 | 0 | 0 |
| PanTT26-P111 | KELRALRKMVSNMSG | KELRALREMVSNMSG | [SPERT](http://www.genecards.org/cgi-bin/carddisp.pl?gene=SPERT) | 145 | 65 | 0 | 0 |
| PanTT26-P112 | AGQNPASHPPPDDAE | AGQNPASDPPPDDAE | [TADA1](http://www.genecards.org/cgi-bin/carddisp.pl?gene=TADA1) | 13 | 10 | 0 | 0 |
| PanTT26-P113 | PTKCEVERFTATSFG | PTKCEVEQFTATSFG | [TG](http://www.genecards.org/cgi-bin/carddisp.pl?gene=TG) | 24 | 17 | 0 | 0 |
| PanTT26-P114 | IHSSWDCGLFTNYSA | IHSSWDCSLFTNYSA | [TMC8](http://www.genecards.org/cgi-bin/carddisp.pl?gene=TMC8) | 38 | 61 | 0 | 408 |
| PanTT26-P115 | IMASKGMRHFCLISE | IMASKGMHHFCLISE | [TMEM168](http://www.genecards.org/cgi-bin/carddisp.pl?gene=TMEM168) | 132 | 92 | 213 | 193 |
| PanTT26-P116 | LWHLQGPKDLMLKLR | LWHLQGPEDLMLKLR | [TMPRSS6](http://www.genecards.org/cgi-bin/carddisp.pl?gene=TMPRSS6) | 275 | 12 | 371 | 0 |
| PanTT26-P117 | GRNSFEVRVCACPGR | GRNSFEVLVCACPGR | [TP53](http://www.genecards.org/cgi-bin/carddisp.pl?gene=TP53) | 92 | 42 | 0 | 0 |
| PanTT26-P118 | TSCARRDDPRASSPN | TSCARRDYPRASSPN | [TRIOBP](http://www.genecards.org/cgi-bin/carddisp.pl?gene=TRIOBP) | 0 | 53 | 0 | 0 |
| PanTT26-P119 | LGLWRGEEVTLSNPK | LGLWRGEAVTLSNPK | [TRIP12](http://www.genecards.org/cgi-bin/carddisp.pl?gene=TRIP12) | 13 | 74 | 0 | 240 |
| PanTT26-P120 | GCLGGENRFRLRLES | GCLGGENCFRLRLES | [TRPM4](http://www.genecards.org/cgi-bin/carddisp.pl?gene=TRPM4) | 0 | 26 | 0 | 7 |
| PanTT26-P121 | TQLRLPGCPTPVSFG | TQLRLPGWPTPVSFG | [VARS2](http://www.genecards.org/cgi-bin/carddisp.pl?gene=VARS2) | 0 | 99 | 0 | 0 |
| PanTT26-P122 | RKFISLHRKALESDF | RKFISLHKKALESDF | [WDFY4](http://www.genecards.org/cgi-bin/carddisp.pl?gene=WDFY4) | 23 | 249 | 0 | 562 |
| PanTT26-P123 | SGSGSGPLPSLFLNS | SGSGSGPFPSLFLNS | [ZFHX3](http://www.genecards.org/cgi-bin/carddisp.pl?gene=ZFHX3) | 76 | 13 | 85 | 65 |
| PanTT26-P124 | GCGKVFARSENLKIH | GCGKVFACSENLKIH | [ZIC1](http://www.genecards.org/cgi-bin/carddisp.pl?gene=ZIC1) | 43 | 0 | 186 | 0 |
| PanTT26-P125 | STLLTEHRRIHTGEK | STLLTEHLRIHTGEK | [ZNF135](http://www.genecards.org/cgi-bin/carddisp.pl?gene=ZNF135) | 0 | 10 | 0 | 0 |
| PanTT26-P126 | EKPYLCPDCGRGFGQ | EKPYLCPECGRGFGQ | [ZNF169](http://www.genecards.org/cgi-bin/carddisp.pl?gene=ZNF169) | 0 | 0 | 0 | 0 |
| PanTT26-P127 | EECGKPFNRFSYLTV | EECGKPFKRFSYLTV | [ZNF257](http://www.genecards.org/cgi-bin/carddisp.pl?gene=ZNF257) | 0 | 80 | 0 | 0 |
| PanTT26-P128 | YECNECGKAFSQSSH | YECNECGNAFSQSSH | [ZNF3](http://www.genecards.org/cgi-bin/carddisp.pl?gene=ZNF3) | 0 | 0 | 0 | 0 |
| PanTT26-P129 | SHNSSLILHQRVHTG | SHNSSLIFHQRVHTG | [ZNF304](http://www.genecards.org/cgi-bin/carddisp.pl?gene=ZNF304) | 0 | 67 | 0 | 0 |
| PanTT26-P130 | VTGGRGGRQGPSPAF | VTGGRGGWQGPSPAF | [ZNF385C](http://www.genecards.org/cgi-bin/carddisp.pl?gene=ZNF385C) | 58 | 82 | 0 | 3 |
| PanTT26-P131 | CNFSTIDVVSLKTDT | CNFSTIDVSLKTDTE | [ZNF407](http://www.genecards.org/cgi-bin/carddisp.pl?gene=ZNF407) | 56 | 40 | 0 | 70 |
| PanTT26-P132 | SNLTKHKKIHIEKKP | SNLTKHKIIHIEKKP | [ZNF43](http://www.genecards.org/cgi-bin/carddisp.pl?gene=ZNF43) | 369 | 170 | 703 | 0 |
| PanTT26-P133 | ECGQAFSLSSNLMRH | ECGQAFSISSNLMRH | [ZNF479](http://www.genecards.org/cgi-bin/carddisp.pl?gene=ZNF479) | 0 | 80 | 0 | 0 |
| PanTT26-P134 | IHKMIHTGEKPYKCE | IHKMIHTVEKPYKCE | [ZNF493](http://www.genecards.org/cgi-bin/carddisp.pl?gene=ZNF493) | 0 | 38 | 19 | 0 |
| PanTT26-P135 | CNECGKAFCQSPSLI | CNECGKALCQSPSLI | [ZNF501](http://www.genecards.org/cgi-bin/carddisp.pl?gene=ZNF501) | 0 | 36 | 0 | 0 |
| PanTT26-P136 | ECGKAFNRSSNLTKH | ECGKAFNSSSNLTKH | [ZNF506](http://www.genecards.org/cgi-bin/carddisp.pl?gene=ZNF506) | 0 | 17 | 0 | 0 |
| PanTT26-P137 | LQNHIQTIHRELVPD | LQNHIQTFHRELVPD | [ZNF521](http://www.genecards.org/cgi-bin/carddisp.pl?gene=ZNF521) | 94 | 71 | 0 | 0 |
| PanTT26-P138 | SNDSSLTQHQRVHTG | SNDSSLTHHQRVHTG | [ZNF570](http://www.genecards.org/cgi-bin/carddisp.pl?gene=ZNF570) | 0 | 52 | 0 | 0 |
| PanTT26-P139 | SNLTTHKKIHTGERP | SNLTTHKIIHTGERP | [ZNF626](http://www.genecards.org/cgi-bin/carddisp.pl?gene=ZNF626) | 66 | 24 | 14 | 109 |
| PanTT26-P140 | NVAKPSSGPHTLLHI | NVAKPSSCPHTLLHI | [ZNF626](http://www.genecards.org/cgi-bin/carddisp.pl?gene=ZNF626) | 80 | 0 | 465 | 0 |
| PanTT26-P141 | STLNTHKRIHTGEEP | STLNTHKSIHTGEEP | [ZNF679](http://www.genecards.org/cgi-bin/carddisp.pl?gene=ZNF679) | 0 | 21 | 0 | 0 |
| PanTT26-P142 | KCDECGNVFNWPATL | KCDECGNDFNWPATL | [ZNF680](http://www.genecards.org/cgi-bin/carddisp.pl?gene=ZNF680) | 0 | 24 | 0 | 0 |
| PanTT26-P143 | CKECGKAFSSSSHLI | CKECGKALSSSSHLI | [ZNF699](http://www.genecards.org/cgi-bin/carddisp.pl?gene=ZNF699) | 0 | 0 | 0 | 0 |
| PanTT26-P144 | HQRTHTGEKPFKCDE | HQRTHTGDKPFKCDE | [ZNF7](http://www.genecards.org/cgi-bin/carddisp.pl?gene=ZNF7) | 0 | 38 | 0 | 0 |
| PanTT26-P145 | EECGKAFSVFSTLTK | EECGKAFRVFSTLTK | [ZNF708](http://www.genecards.org/cgi-bin/carddisp.pl?gene=ZNF708) | 12 | 70 | 0 | 0 |
| PanTT26-P146 | HKRIHNGEKPYKCEE | HKRIHNGDKPYKCEE | [ZNF730](http://www.genecards.org/cgi-bin/carddisp.pl?gene=ZNF730) | 0 | 137 | 0 | 298 |
| PanTT26-P147 | EKPYSCPDCSLRFAY | EKPYSCPECSLRFAY | [ZNF785](http://www.genecards.org/cgi-bin/carddisp.pl?gene=ZNF785) | 94 | 15 | 159 | 0 |
| PanTT26-P148 | KCEECDTVFSRKSHH | KCEECDTDFSRKSHH | [ZNF860](http://www.genecards.org/cgi-bin/carddisp.pl?gene=ZNF860) | 47 | 28 | 396 | 0 |
| PanTT26-P149 | KAFSQSSTLRKHEII | KAFSQSSSLRKHEII | [ZNF99](http://www.genecards.org/cgi-bin/carddisp.pl?gene=ZNF99) | 0 | 43 | 0 | 0 |

Legend: ATCL = autologous tumour cell line

**Supplementary Table 2**

| **Patient ID** | **HLA class/type** | **Location of mutation in protein** | **Wildtype sequence** | **Mutated sequence** |
| --- | --- | --- | --- | --- |
| PanTT26 | [HLA-DRB1](http://www.genecards.org/cgi-bin/carddisp.pl?gene=HLA-DRB1) | A102G | DILEQAR**A**AVDTYCR | DILEQAR**G**AVDTYCR |
| PanTT39 | HLA-A | G10W | MYGCDVGSD**G**RFLRGYRQDAYD | MYGCDVGSD**W**RFLRGYRQDAYD |
|  | HLA-A | G131W | IQIMYGCDVGPD**G**RFLRGYRQDAYD | IQIMYGCDVGPD**W**RFLRGYRQDAYD |
|  | HLA-A | G131W | IQIMYGCDVGSD**G**RFLRGYRQDAYD | IQIMYGCDVGSD**W**RFLRGYRQDAYD |
|  | HLA-A | R138Q | DVGPDGRFLRGY**R**QDAYDGKDYIAL | DVGPDGRFLRGY**Q**QDAYDGKDYIAL |
|  | HLA-A | R138Q | DVGSDGRFLRGY**R**QDAYDGKDYIAL | DVGSDGRFLRGY**Q**QDAYDGKDYIAL |
|  | HLA-A | D140Y | GSDGRFLRGYRQ**D**AYDGKDYIALNE | GSDGRFLRGYRQ**Y**AYDGKDYIALNE |
|  | HLA-A | D140Y | GPDGRFLRGYRQ**D**AYDGKDYIALNE | GPDGRFLRGYRQ**Y**AYDGKDYIALNE |
|  | HLA-B | Y167H | YLEGECVEWLRR**Y**LENGKDKLERAG | YLEGECVEWLRR**H**LENGKDKLERAG |
|  | HLA-B | Y195H | YLEGECVEWLRR**Y**LENGKDKLERAD | YLEGECVEWLRR**H**LENGKDKLERAD |
|  | HLA-C | R30K | ALTETWACSHSM**R**YFDTAVSRPGRG | ALTETWACSHSM**K**YFDTAVSRPGRG |
|  | HLA-C | R2K | M**R**YFDTAVSRPGRG | M**K**YFDTAVSRPGRG |
|  | HLA-C | D5V | MRYF**D**TAVSRPGRGEPR | MRYF**V**TAVSRPGRGEPR |
|  | HLA-C | D5Y | MRYF**D**TAVSRPGRGEPR | MRYF**Y**TAVSRPGRGEPR |
|  | HLA-C | A7S | MRYFDT**A**VSRPGRGEPRFI | MRYFDT**S**VSRPGRGEPRFI |
|  | HLA-C | D33Y | ETWACSHSMRYF**D**TAVSRPGRGEPR | ETWACSHSMRYF**Y**TAVSRPGRGEPR |
|  | HLA-C | D33V | ETWACSHSMRYF**D**TAVSRPGRGEPR | ETWACSHSMRYF**V**TAVSRPGRGEPR |
|  | HLA-C | A35S | WACSHSMRYFDT**A**VSRPGRGEPRFI | WACSHSMRYFDT**S**VSRPGRGEPRFI |
|  | HLA-DRB1 | T92R | FRNQKGHSGLQP**T**GNTF | FRNQKGHSGLQP**R**GNTF |
|  | HLA-DRB1 | T262R | FRNQKGHSGLQP**T**GFLS | FRNQKGHSGLQP**R**GFLS |
|  | HLA-DPA1 | A42V | GAIKADHVSTYA**A**FVQTHRPTGEFM | GAIKADHVSTYA**V**FVQTHRPTGEFM |
|  | HLA-DPA1 | A42T | GAIKADHVSTYA**A**FVQTHRPTGEFM | GAIKADHVSTYA**T**FVQTHRPTGEFM |

Legend: WT = wildtype; Mut = mutated

**Supplementary Table 3**

| **Peptide ID** | **Gene name** | **Sequence (wildtype)** | **Sequence (mutated)** | **HLA class II-restricting element** |
| --- | --- | --- | --- | --- |
| PanTT39-P1 | DOCK3 | AFT**L**LLYCELLQWED | AFT**M**LLYCELLQWED | HLA-DQA10101-DQB10201 |
| PanTT39-P2 | AQP7 | TIYSLFYSVAD**R**DAPA | TIYSLFYSVAD**Q**DAPA | HLA-DQA10501-DQB10501 |
| PanTT39-P3 | CCDC39 | FKQDLM**I**EDNLL | FKQDLM**L**EDNLL | HLA-DQA10101-DQB10201 |
| PanTT39-P4 | CCDC39 | QDLM**I**EDNLLKLEV | QDLM**L**EDNLLKLEV | DRB1-0301 |
| PanTT39-P5 | K7N7A8 | GLLR**D**WRTERLF | GLLR**Y**WRTERLF | DRB1-0301 |
| PanTT39-P6 | K7N7A8 | ILFSLQPGLLR**D**W | ILFSLQPGLLR**Y**W | DRB1-0101 |
| PanTT39-P7 | TENM3 | NVSFFHY**P**EYGY | NVSFFHY**Q**EYGY | HLA-DQA10101-DQB10501 |
| PanTT39-P8 | IPO8 | EFPV**R**QAAAIYLK | EFPV**L**QAAAIYLK | DRB1-0101 |
| PanTT39-P9 | CFTR | INFKIERGQ**L**LAV | INFKIERGQLAV | DRB1-0101 |
| PanTT39-P10 | VPS4B | GAIVIE**R**PNVKWS | GAIVIE**L**PNVKWS | DRB1-0301 |
| PanTT39-P11 | MORC1 | LNKVTIDA**R**HRLPL | LNKVTIDA**I**HRLPL | DRB1-0301 |
| PanTT39-P12 | CDKL3 | DFGFA**R**TLAAPGDI | DFGFA**L**TLAAPGDI | DRB1-0101 |
| PanTT39-P13 | NUP93 | LEL**M**NKLLSPVVPQ | LEL**I**NKLLSPVVPQ | DRB1-0101 |
| PanTT39-P14 | SPTA1 | IEEL**R**HLWDLLLELTL | IEEL**H**HLWDLLLELTL | HLA-DQA10101-DQB10201 |
