# Supplementary figures and images for: Neoepitope targets of tumour-infiltrating lymphocytes from patients with pancreatic cancer
Source: Br J Cancer. 2018 Oct 31;120(1):97–108. doi: 10.1038/s41416-018-0262-z (PMC6325142; doi:10.1038/s41416-018-0262-z)

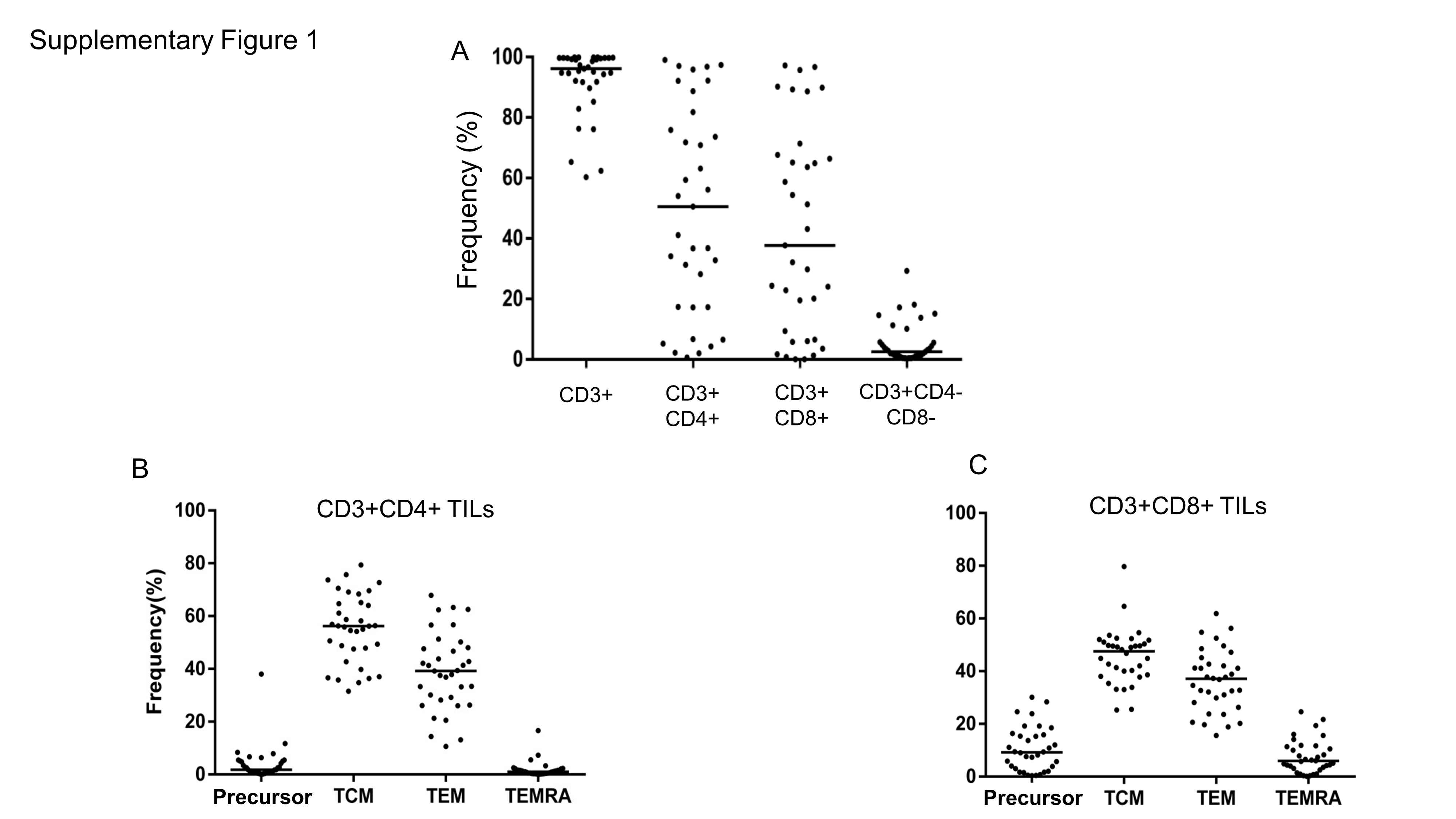

Supplement: Supplementary file 2 — Supplementary Figure S1 [file 41416_2018_262_MOESM2_ESM.tif]

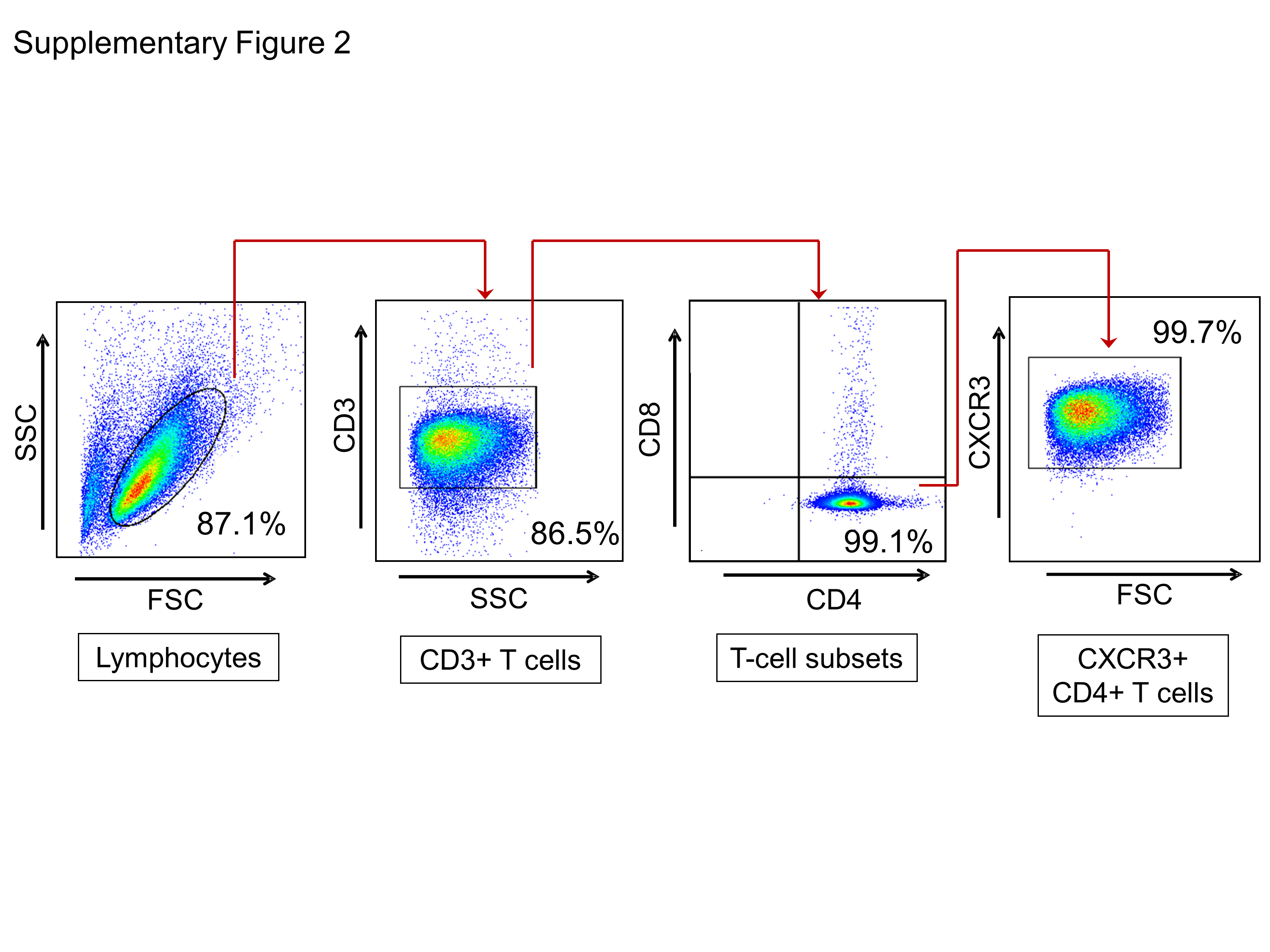

Supplement: Supplementary file 3 — Supplementary Figure S2 [file 41416_2018_262_MOESM3_ESM.tif]

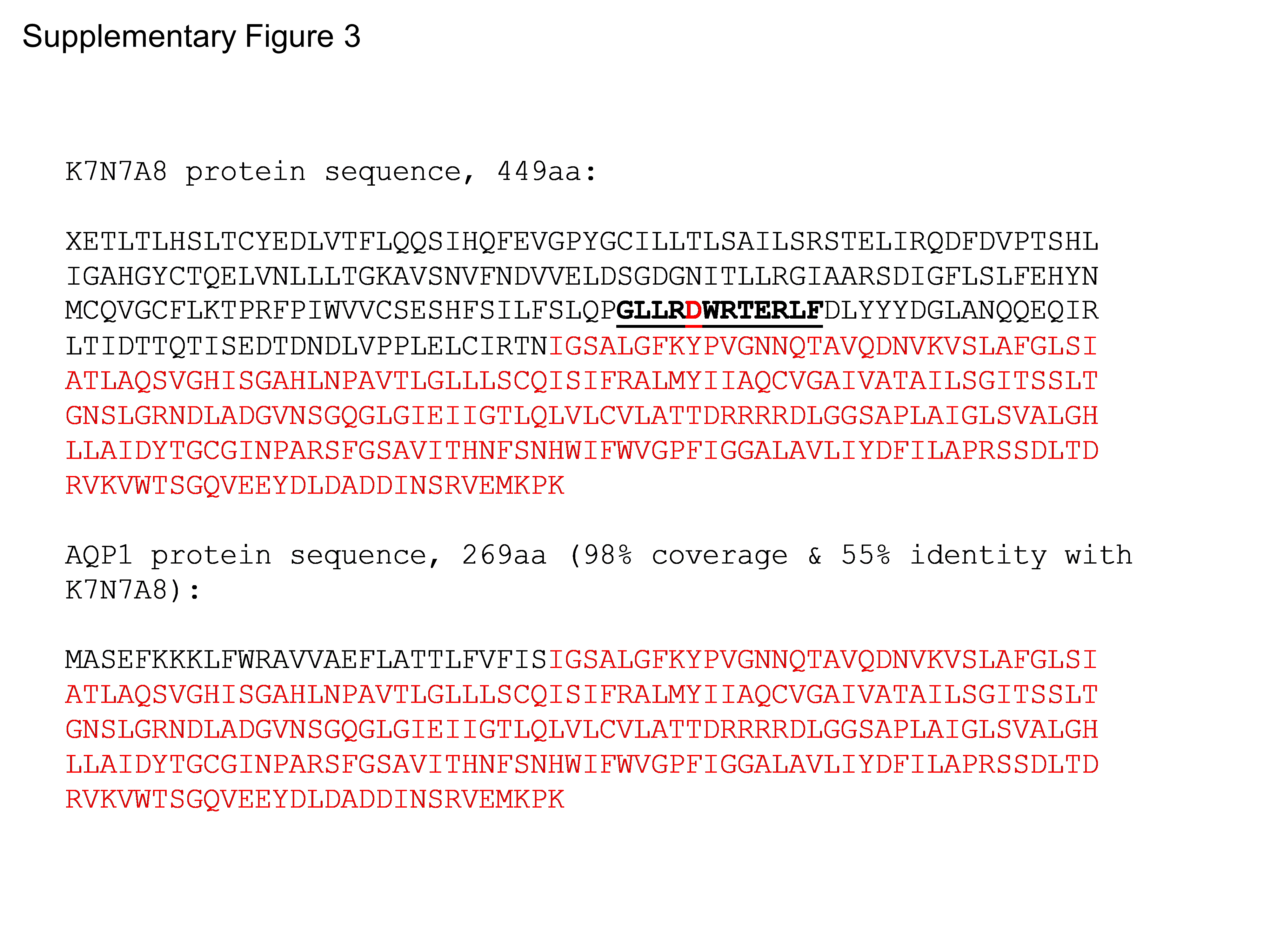

Supplement: Supplementary file 4 — Supplementary Figure S3 [file 41416_2018_262_MOESM4_ESM.tif]
